# Advancing shock prediction: leveraging prior knowledge and self-controlled data for enhanced model accuracy and generalizability

**DOI:** 10.1186/s12911-025-03108-2

**Published:** 2025-07-14

**Authors:** Cheng-Yu Tsai, Xiu-Rong Huang, Po-Tsun Kuo, Tzu-Tao Chen, Yun-Kai Yeh, Kuan-Yuan Chen, Arnab Majumdar, Chien-Hua Tseng

**Affiliations:** 1 Division of Pulmonary Medicine, Department of Internal Medicine, Taipei Medical University-Shuang Ho Hospital, New Taipei City, 235041 Taiwan; 2 School of Respiratory Therapy, College of Medicine, Taipei Medical University, Taipei, 110301 Taiwan; 3 Sleep Center, Taipei Medical University-Shuang Ho Hospital, New Taipei City, Taiwan; 4 TMU Research Center of Artificial Intelligence in Medicine and Health, Taipei Medical University, Taipei, Taiwan; 5 Graduate Institute of Nanomedicine and Medical Engineering, College of Biomedical Engineering, Taipei Medical University, Taipei, Taiwan; 6 Advantech Co., Ltd., Taipei, Taiwan; 7 College of AI, National Yang Ming Chiao Tung University, Hsinchu, Taiwan; 8 Division of Critical Care Medicine, Department of Emergency and Critical Care Medicine, Shuang Ho Hospital, Taipei Medical University, No.291, Zhongzheng Rd., Zhonghe District, New Taipei City, 235041 Taiwan; 9 Division of Pulmonary Medicine, Department of Internal Medicine, School of Medicine, College of Medicine, Taipei Medical University, Taipei, Taiwan; 10 Department of Civil and Environmental Engineering, Imperial College London, London, SW7 2AZ UK

**Keywords:** Shock, Early identification models, Machine learning, Feature engineering, Medical information Mart for intensive care III (MIMIC-3)

## Abstract

**Objectives:**

Timely intervention in shock is vital, as delays over one hour greatly increase mortality. This study aims to develop an enhanced machine learning model that improves predictive performance by utilizing self-controlled data and applying feature engineering informed by medical knowledge to physiological waveforms, enabling the prediction of shock one hour in advance without relying on blood tests.

**Methods:**

Patient data and physiological waveforms were obtained from the Medical Information Mart for Intensive Care III (MIMIC-3) database. Shock was defined as a mean arterial pressure ≤ 65 mmHg for more than one minute, combined with serum lactate levels ≥ 2 mmol/L within 12 h before or after the hypotension event. Waveforms used for prediction were extracted from 30 min time-segment before a 1-hour period prior to the event. Self-controlled waveforms were obtained from the same patient either one day before or up to seven days after the shock event.

**Results:**

The study included 389 ICU patients who met the shock criteria and had complete physiological waveform data available for analysis. A total of 299 features were derived: 90 from arterial blood pressure (ABP), 89 from electrocardiogram (ECG), 112 from respiratory waveforms (RESP), and 8 from blood oxygen saturation (SpO_2_). The weighted ensemble model showed the best performance with an AUC of 0.93 and accuracy of 84.15%, and sensitivity of 79.64% in the testing set. The most predictive features included ECG_HRV_pNN50 (proportion of successive heartbeat intervals differing by more than 50 ms), RESP_Width_Mean (mean width of respiratory waveform), RESP_Cycle_Rate_Mean (mean respiratory cycle rate), ABP_TimeSBP2DBP_SampEn (sample entropy of systolic-diastolic intervals), and ABP_AmplitudeDBP_Median (median amplitude of diastolic peaks).

**Conclusions:**

This study demonstrated the feasibility of predicting shock one hour before its onset using only four physiological waveforms, combined with feature engineering based on physiological concepts and self-sampling data. The model achieved a strong AUC and a high sensitivity.

**Clinical trial number:**

Not applicable.

**Supplementary Information:**

The online version contains supplementary material available at 10.1186/s12911-025-03108-2.

## Introduction

Untreated and progressively worsening shock leads to an increasingly higher mortality rate [1]. Early detection, particularly within the first hour, followed by prompt intervention, can significantly reduce the mortality rate [2]. Consequently, the development of an hourly adaptive hypotension prediction model holds substantial clinical value. Most existing risk models incorporate blood tests [3], which cannot be performed every hour. However, if we can predict shock risk by analyzing four continuously monitored physiological waveforms—arterial blood pressure (ABP), electrocardiogram (ECG), respiratory waveform, and blood oxygen waveform—without the need for blood draws, it would enable minute-by-minute risk updates and provide real-time dynamic predictions.

A previous study employed a machine learning algorithm to analyze thousands of features for predicting acute hypotension, achieving a sensitivity of 88% and a specificity of 87% 15 min before onset, with predictive performance improving closer to the event [4]. Yoon et al. directly utilized vital signs waveform data to predict hypotension events in the intensive care unit (ICU) at least 1 h before the initial episode, achieving a high area under the receiver operating characteristic curve (AUC) of 0.88. However, the positive predictive value was only around 57.7–65.2% [5], leading to a high rate of false alarms, which can cause alarm fatigue, where healthcare providers become desensitized to alarms. By analyzing the waveform data using pathophysiological background concepts, the model can be based on clinical meaningful features, thereby enhancing its generalizability to other populations [6]. Time-domain features, such as intervals between systolic and diastolic peaks, are linked to myocardial contractility and cardiovascular dysfunction, which are relevant for shock progression [7]. Shock is also associated with hypercapnia [8]. Subtle changes in respiratory waveform patterns, such as rise-decay and peak-trough symmetry, can indicate hemodynamic distress [9]. Variations in heart and respiratory rates have been suggested as indicators of organ dysfunction, allowing for accurate screening of septic shock risk [10]. Deriving features based on prior knowledge from vital signs waveforms not only improves interpretability but also enhances generalizability. To further improve generalizability, it is important to consider that due to individual variations, constructing models by comparing waveforms to individual baselines, rather than to those of others, can more accurately identify features related to the occurrence of shock [11].

In this study, we defined shock as the concurrent presence of hypotension and elevated lactate levels to ensure strict and unambiguous case identification. We employed four routinely recorded ICU waveforms—arterial blood pressure, ECG, respiratory movement, and pulse oximetry—eschewing blood draws to enhance clinical applicability and reduce invasive sampling. By using non-shock periods within the same patients as control signals and incorporating physiological insights into waveform analysis, we aim to predict shock onset one hour in advance with improved specificity and without requiring additional blood tests.

## Materials and methods

### Data source

The dataset utilized in this study is derived from an open-source clinical database, the Medical Information Mart for Intensive Care III (MIMIC-III, version 1.3, released on December 10, 2015) [12]. This retrospective dataset, encompassing diverse granularity levels, was compiled from a tertiary care hospital in Boston, Massachusetts, over the period from 2000 to 2014. To identify shock events, all patients in the database were screened using the inclusion criteria of a mean arterial pressure (MAP) ≤ 65 mmHg for more than 1 min and serum lactate levels > 2 mmol/L within 12 h before or after the hypotension event [13]. This 12-hour window is selected to account for the irregular and infrequent timing of lactate measurements in retrospective clinical datasets. Notably, only the first shock event for each patient was included in the study, ensuring that each patient contributed equally to the analysis and thereby avoiding potential confounding effects. For the eligible subjects, their physiological data, including blood pressure, ECG, respiratory waveforms, and blood oxygen waveform were used to calculate other relevant features for establishing models. Patients lacking any of the four vital signs waveform datasets, those with data timeframes shorter than 90 min prior to the shock event, or those with more than 50% missing values were excluded.

### Shock data determination, data pre-processing and feature engineering

**Fig. 1:**
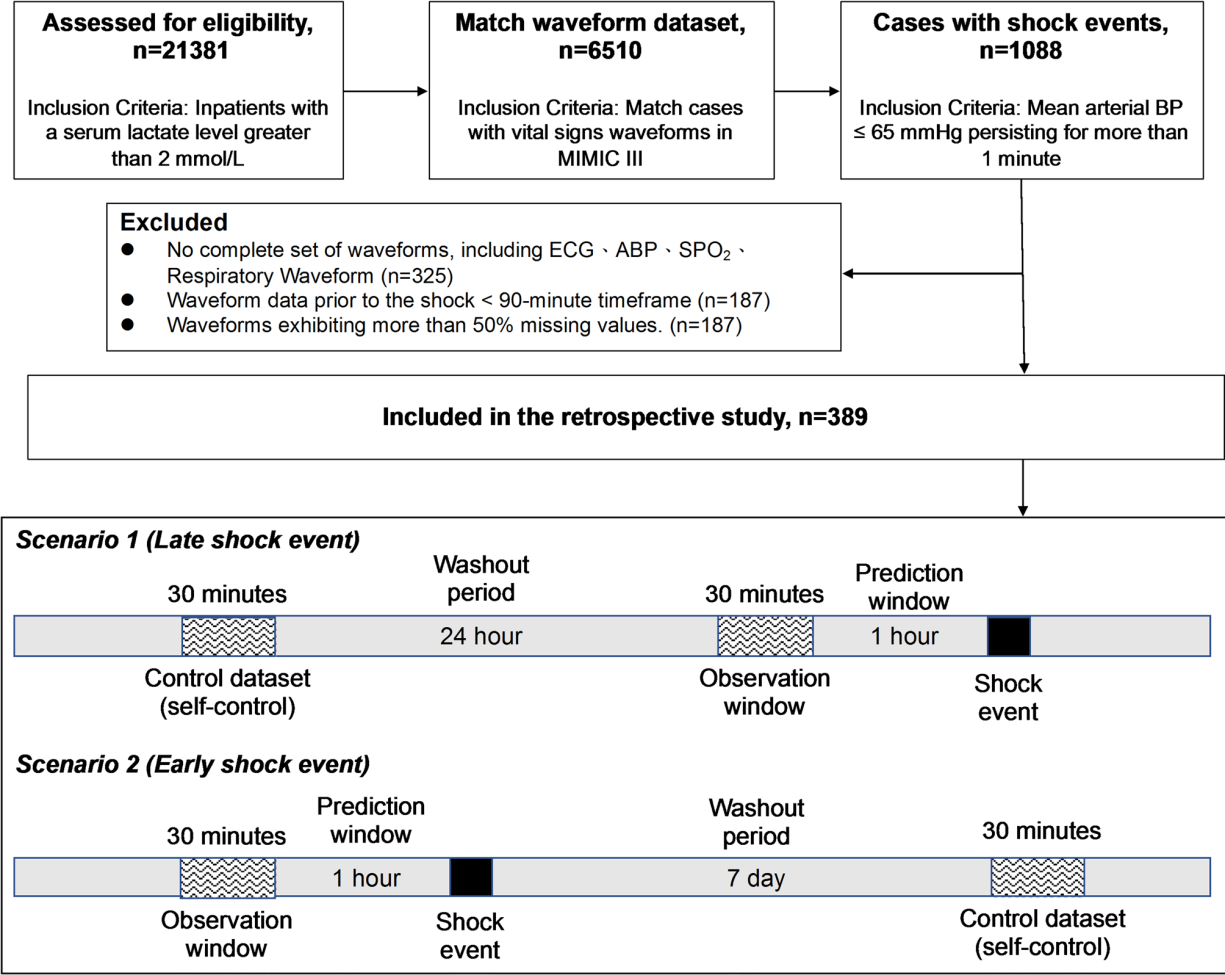
Data Collection procedure. A total of 389 participants were included. For each shock event, a 30-minute observation window was extracted one hour prior to the event. Self-control data were obtained from the same patients using two washout strategies to minimize potential physiological influence on the control data: (1) Scenario 1– a 30-minute control segment collected at least 24 hours before the shock event; (2) Scenario 2– a 30-minute control segment collected 7 days after the event. These strategies ensured clear separation between shock and non-shock states for model development. Abbreviations: BP: Blood Pressure; ECG: Electrocardiogram; ABP: Arterial Blood Pressure; SpO2: Peripheral Capillary Oxygen Saturation

The timeline for data collection and analysis in the study includes several phases: first, the shock event is identified (Fig. 1). Second, a 1-hour prediction window is designated to predict the occurrence of the shock event. Before this prediction window, a 30-minute observation window is used to collect waveform data to predict the impending shock event. Finally, normal (non-shock) data are derived from the same patients and served as self-control samples. To minimize the potential influence of shock-related physiological changes on these control data, two types of washout periods were applied depending on the study scenario. In Scenario 1 (late shock event), a 24-hour washout period is implemented prior to the shock event, and a 30-minute control dataset is collected before this washout period. In Scenario 2 (early shock event), a 7-day washout period is applied after the shock event, and a 30-minute control dataset is collected at the end of this period. These washout strategies aimed to ensure a clearer physiological separation between shock and non-shock data, improving the validity of model training and evaluation.

A 10-minute rolling window with a 5-minute overlap was used to extract physiological signals, with features captured from each 10-minute interval for feature creation [14, 15]. The Multivariate Imputation by Chained Equations (MICE) algorithm was employed to address missing values in the physiological waveform data [16]. For the MICE-processed ECG data, this study filtered them through a passband (ranging between 3 and 45 Hz) and used to calculate heart rate variability (HRV), including time-, frequency-, and nonlinear-domain features [17]. Besides, peak interpolation was applied for heart rates below 40 bpm or above 150 bpm (400 milliseconds < R-R intervals < 1500 milliseconds) to partially address the peak-picking issue associated with turning-point selection [18]. All derived RRI values were further manually reviewed to ensure signal reliability and minimize potential errors in subsequent HRV-based modeling.

For the MICE-processed blood pressure waveform data, this study then employed the signal quality index algorithm to remove outliers (check window: 0.5 s) and calculated statistical features, such as mean, median, skewness, and entropy [19, 20]. For the MICE-processed respiratory waveform, this study initially employed the optimized breath detection algorithm proposed by previous researchers to identify each breathing pattern [21]. Next, statistical features for the time domain, such as interval mean, interval median, and skewness, were extracted [22]. Additionally, geometric features such as respiratory volume per time, rise-decay symmetry, and peak-trough symmetry were obtained, and respiratory rate variability was derived [23–25]. Similarly, for the MICE-processed blood oxygen waveform data, this study then determined the statistical features, such as mean, median, standard deviation, and entropy [26]. Following the feature engineering process, mutual information-based feature selection was applied to identify the top 65 features most relevant to shock events. The selection of 65 features was based on an iterative testing process, systematically evaluating feature subsets ranging from 30 (approximately 1/10 of the total features) to 100 (approximately 1/3 of the total features) based on their mutual information ranking. Next, this study independently partitioned the data based on a unique patient number, dividing it into three datasets: a training set (60%), a validation set (20%), and a testing set (20%). This method ensured that data extracted from a single patient did not dominantly or repeatedly appear in more than one set, thereby enhancing the credibility and reproducibility of the established models. In the training and validation phases, mutual information was calculated for all derived features to estimate predictability based on their entropy [27].

### Model establishment and feature importance

**Fig. 2:**
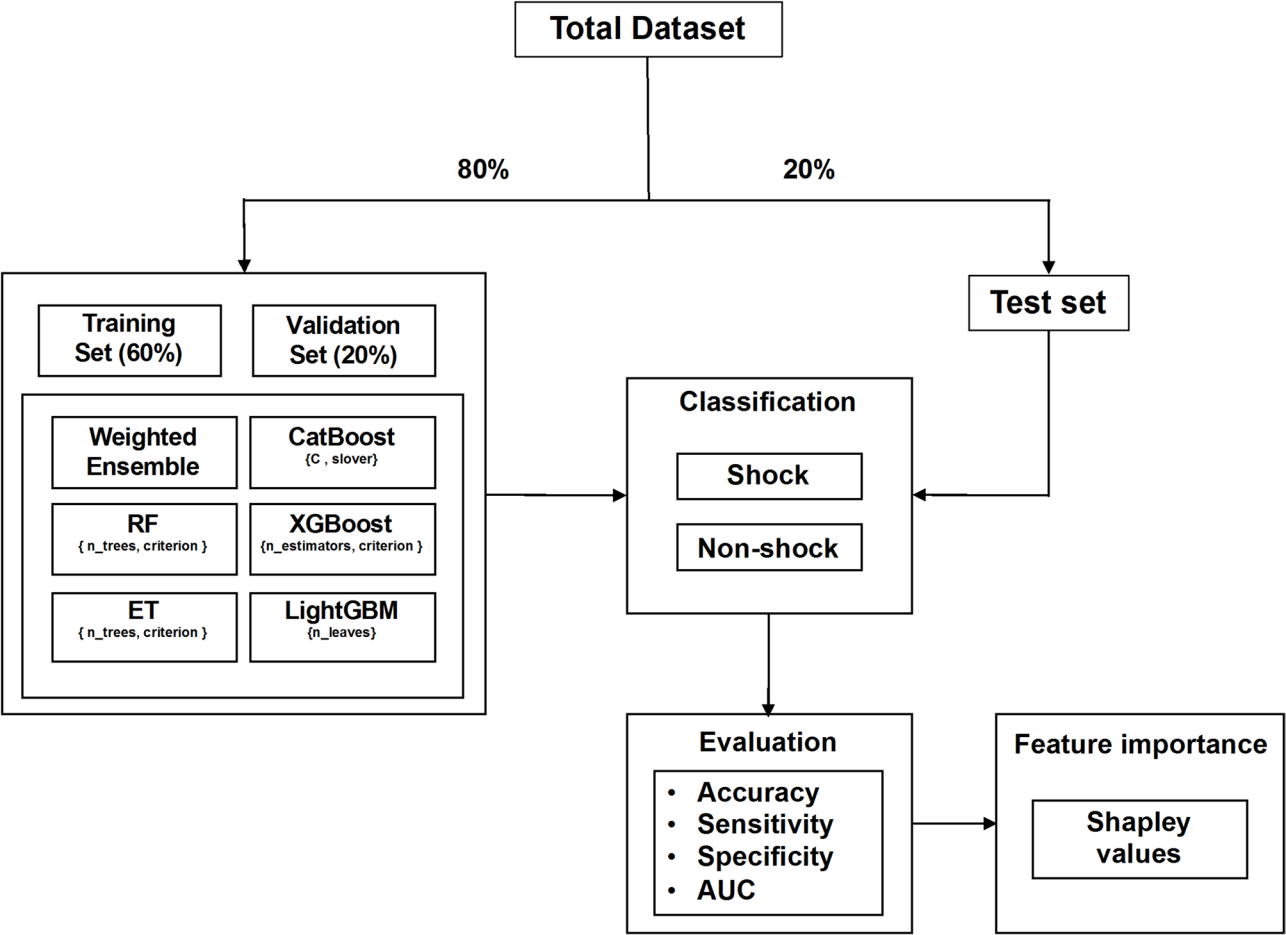
Model establishment and evaluation scheme. The dataset was split into training-validation and testing sets using an 80:20 ratio. Machine learning models developed included weighted ensemble, extreme gradient boosting (XGBoost), categorical boosting (CatBoost), light gradient boosting machine (LightGBM), random forest (RF), and extremely randomized trees (ET). Model performance was evaluated using accuracy, area under the receiver operating characteristic curve (AUC), sensitivity, and specificity. Shapley values from the best-performing model were used to interpret feature importance. Abbreviations: weighted ensemble: constructing ensembles from all models

This study utilized the artificial intelligence framework service platform (WISE-PaaS/AIFS, version 3.8.11, Advantech Co., Ltd., Neihu District, Taipei City, Taiwan) to develop the models. The model development and evaluation process is illustrated in Fig. 2. Six types of models were established in the current study, including weighted ensemble, extreme gradient boosting (XGBoost), categorical boosting (CatBoost), light gradient boosting machine (LightGBM), random forest (RF), and extremely randomized trees (ET). These models have been employed in predicting various diseases based on physiological signals [28–30]. Regarding the hyperparameters, the weighted ensemble consisted of various models [31]. Next, the logistic regression with L2 regularization was the default setting in XGBoost and CatBoost, both configured with a maximum depth of 6, and in LightGBM, which was configured with 31 leaves [32]. For RF and ET, the number of estimators was set at 100, using the Gini index as the classification criterion [33]. Regarding model performances, this study evaluated accuracy, AUC, sensitivity, and specificity, as these metrics are more clinically relevant and appropriate for the study design. Shapley values of the best-performing model are used to represent feature importance [34]. Simple logistic regression models are employed to confirm the significant parameters. All statistical analyses were conducted using Scikit-learn (version 0.21.2, Python Software Foundation, Fredericksburg, VA, USA). Multivariable logistic regression, adjusted for age, sex, body mass index (BMI), and neck and waist circumference, was utilized to explore the relationship between shock and non-shock occurrences. The level of statistical significance was set to a two-sided threshold of *P* < 0.05.

## Results

### Baseline and disease characteristics

From the MIMIC-III dataset, 21,381 cases met the criteria with a serum lactate level greater than 2 mmol/L, and we were able to assess 6,510 cases with vital sign waveforms. We then identified 1,088 participants with shock occurrence. Cases with no complete set of four waveforms (*n* = 325), no waveform data prior to shock (*n* = 187), or more than 50% missing values (*n* = 187) were excluded. Ultimately, 389 ICU patients who experienced shock were included in the analysis (Table 1). The average age of the participants was 65.04 years (SD: 14.82), with a gender distribution of 184 males and 163 females. The average BMI was 29.64 kg/m² (SD: 7.39). Among underlying diseases, 39.59% of participants had congestive heart failure, 31.36% had diabetes mellitus, and 30.07% had mild liver disease. Chronic obstructive pulmonary disease was present in 28.28% of participants, and liver disease in 26.22%. Additionally, 24.68% had cerebrovascular disease, 21.85% had a history of myocardial infarction, and 15.68% had renal disease (Table 1).

**Table 1:** Baseline characteristics of enrolled participants

Patient characteristics	Mean ± SD (%)
Age (years)	65.04 ± 14.82
Sex (male/female)	184 / 163
Height	169.11 ± 10.62
Weight	85.08 ± 22.07
Body mass index (kg/m^2^)	29.64 ± 7.39
**Underlying disease**	**No. of cases (%)**
Congestive heart failure	154 (39.59%)
Diabetes mellitus	122 (31.36%)
Mild liver disease	117 (30.07%)
Chronic obstructive pulmonary disease	110 (28.28%)
Liver disease	102 (26.22%)
Cerebrovascular disease	96 (24.68%)
Myocardial infarction	85 (21.85%)
Renal disease	61 (15.68%)
Peripheral vascular disease	57 (14.65%)
Malignancy	46 (11.83%)
Diabetes mellitus with chronic complications	37 (9.51%)
Metastatic solid tumor	28 (7.2%)
Peptic ulcer disease	21 (5.4%)
Rheumatic disease	10 (2.57%)
Dementia	7 (1.8%)
Hemiplegia	4 (1.02%)

Abbreviation: SD, standard deviation

### Parameters derived from vital signs waveforms

A total of 299 features were derived from ABP, ECG, respiratory waveforms, and blood oxygen saturation (SpO_2_) signals to predict shock occurrence. The 90 features related to ABP waveforms were generated by combining nine key physiological parameters—systolic BP, diastolic BP, pulse pressure (PP), mean pressure (MeanAP, the average pressure between adjacent onsets), MAP, heart rate (HR), time intervals between systolic and diastolic peaks (TimeSBP2DBP), and difference amplitudes between consecutive systolic (AmplitudeSBP) and diastolic peaks (AmplitudeDBP)—with ten statistical methods: minimum, mean, maximum, median, standard deviation (STD), skewness, kurtosis, Hurst exponent, Lyapunov exponent, and sample entropy (SampEn). This approach produced 90 distinct features, 56 of which (62.2%) were found to be statistically significant in predicting the occurrence of shock in simple logistic regression (Supplementary Table 1).

The 89 features related to ECG waveforms were generated by HRV indices across several analysis domains [17]. Supplementary Fig. 1 presents the distribution of processed R-R intervals derived from HR signals. In the time-domain, indices include deviation-based measures like SDNN (Standard Deviation of NN intervals) and SDANN (Standard Deviation of Average NN intervals), as well as difference-based measures such as RMSSD (Root Mean Square of Successive Differences) and pNN50 (Proportion of NN intervals differing by more than 50 ms). The frequency-domain covers absolute power metrics like ULF (Ultra-Low Frequency), VLF (Very Low Frequency), LF (Low Frequency), and HF (High Frequency), along with normalized power measures such as LnHF (Natural Logarithm of HF) and LF/HF (Ratio of LF to HF power). The time-frequency domain includes methods like STFT (Short-Term Fourier Transform) and WT (Wavelet Transform) for analyzing power spectra over time. Lastly, the non-linear domain features Poincaré plot analysis (e.g., SD1 and SD2), entropy measures (e.g., ApEn, SampEn, and MSE), and fractal dimensions (e.g., DFA and CD) to assess the complexity and self-similarity of HRV signals [17]. Among these, 32 out of 89 (35.9%) electrocardiogram-related features were significant related to shock events (Supplementary Table 2).

A total of 112 features related to respiratory waveforms were derived by applying ten statistical methods—minimum, mean, maximum, median, standard deviation (STD), skewness, kurtosis, Hurst exponent, Lyapunov exponent, and sample entropy (SampEn)—to nine key physiological parameters, including amplitudes between peaks and troughs, time intervals between peaks and troughs, time intervals between peaks, time intervals between troughs, amplitude, rate, respiratory volume per time (RVT), symmetry of peaks and troughs, and symmetry of rise and decay phases [23, 24]. Respiratory rate variability (RRV) was assessed using time domain parameters (e.g., SDBB, RMSSD, SDSD), frequency domain parameters (e.g., PSD, band power, peak frequency) via Burg, Welch, and Lomb–Scargle methods, and nonlinear metrics (e.g., sample entropy, Poincare plot) along with time-frequency measures (e.g., LF-HF ratio) using wavelet analysis [25]. 57 out of 112 (50.9%) respiratory-related features were significant related to shock events (Supplementary Table 3).

For SpO_2_ signals, a total of eight features were extracted. The linear analysis includes five features: the minimum (Smin), mean (Smean), maximum (Smax), median (Smedian), and standard deviation (Ssd) of the SpO_2_ signal. The non-linear analysis considers three features: sample entropy (SampEn), Lempel-Ziv complexity (LZC), and the Central Tendency Measure (CTM). 5 out of 8 oxygen-related features (62.5%) were significant related to shock events (Supplementary Table 4).

### Model performance and feature importance

Using mutual information-based feature selection, we identified the top 65 features most relevant to the shock prediction model, achieving optimal performance. These optimized models, utilizing the selected features, were then applied to classify shock patients in the testing set, enabling an evaluation of their predictive performance. The models employed for shock prediction included Weighted Ensemble, XGBoost, CatBoost, LightGBM, RF, and ET. The Weighted Ensemble model achieved the highest accuracy of 84.97% and AUC of 0.91 in the training and validation set. In the testing set, it maintained an accuracy of 84.15%, the highest AUC of 0.93, a sensitivity of 79.64%, and a specificity of 86%, which corresponds to the highest predictive performance among all models. Other models also demonstrated strong performance; for example, LightGBM achieved an accuracy of 80.98%, an AUC of 0.91, and a sensitivity of 74.37%, while RF achieved an accuracy of 82.29%, an AUC of 0.92, and a sensitivity of 77.87%. These results demonstrate the overall robustness of the models in predicting shock cases (Table 2). The detailed confusion matrices for the established models are presented in supplementary Table 5.

**Table 2:** Comparison of model performance in training, validation and testing sets

Variables	Weighted_Ensemble	CatBoost	LightGBM	RF	XGBoost	ET
**Performance: Training and validation set**						
Accuracy (%)	84.97 (82.51, 87.43)	83.06 (80.60, 85.52)	84.06 (81.54, 86.58)	83.01 (80.73, 85.29)	82.74 (80.02, 85.46)	80.66 (79.54, 81.78)
Sensitivity (%)	84.0 (77.44, 90.56)	82.46 (77.40, 87.52)	83.54 (77.40, 89.68)	80.69 (74.73, 86.65)	82.29 (77.81, 86.77)	77.54 (72.48, 82.60)
Specificity (%)	89.31 (79.05, 99.57)	88.28 (76.22, 100.34)	88.33 (76.11, 100.55)	87.27 (77.31, 97.23)	88.19 (79.35, 97.03)	87.41 (77.87, 96.95)
AUC	0.91 (0.88, 0.94)	0.90 (0.87, 0.93)	0.91 (0.89, 0.93)	0.90 (0.88, 0.93)	0.90 (0.87, 0.93)	0.88 (0.86, 0.90)
**Performance: Testing set**						
Accuracy (%)	84.15 (80.59, 87.71)	82.95 (76.91, 88.99)	80.98 (76.76, 85.20)	82.29 (79.29, 85.29)	83.47 (79.95, 86.99)	81.86 (78.30, 85.42)
Sensitivity (%)	79.64 (65.34, 93.94)	78.50 (67.52, 89.48)	74.37 (63.95, 84.79)	77.87 (65.29, 90.45)	79.30 (65.10, 93.50)	77.07 (64.13, 90.01)
Specificity (%)	86.00 (81.54, 90.46)	83.69 (77.63, 89.75)	84.62 (80.70, 88.54)	85.47 (79.71, 91.23)	83.35 (79.19, 87.51)	83.93 (78.81, 89.05)
AUC	0.93 (0.89, 0.97)	0.92 (0.86, 0.98)	0.91 (0.84, 0.97)	0.92 (0.87, 0.96)	0.93 (0.90, 0.96)	0.91 (0.87, 0.96)

Abbreviation: Weighted_Ensemble: Constructing ensembles from all models; CatBoost: Categorical Boosting; LightGBM: Light gradient boosting machine; RF: Random Forest; XGBoost: Extreme gradient boosting; ET: Extremely randomized trees; AUC: Area under the receiver operating characteristic curve

Data are expressed as the mean (95% confidence interval)

**Fig. 3:**
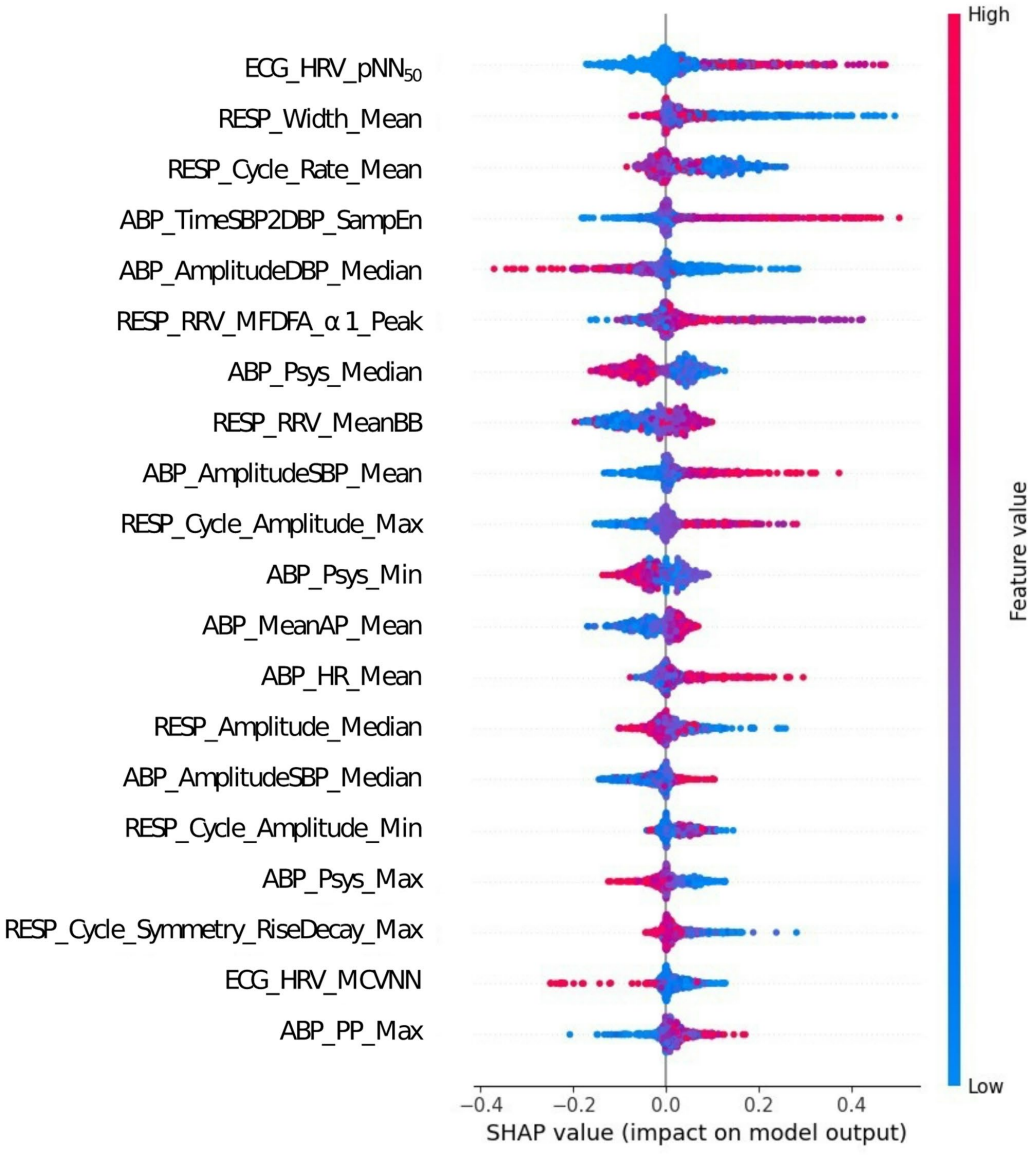
Density scatter plot of Shapley values representing feature importance in the best-performing model. The x-axis represents the Shapley value of each feature, indicating its impact on model output. The y-axis lists input features in ascending order of their mean absolute Shapley values, reflecting their relative importance. Color denotes the original feature value, with red indicating higher values and blue indicating lower values. Abbreviations: ECG, Electrocardiogram; HRV, Heart rate variability; pNN50, Proportion of successive heartbeat intervals that differ by more than 50 milliseconds; RESP, Respiratory waveform; ABP, Arterial blood pressure; TimeSBP2DBP, Time interval between systolic peak and subsequent diastolic peak; SampEn, Sample entropy; RRV, Respiratory rate Variability; MFDFA, Multifractal detrended fluctuation analysis; Psys, Systolic blood pressure; MeanBB, The mean value of the breath-to-breath intervals; AmplitudeSBP, amplitudes between consecutive diastolic peaks; MeanAP, Average pressure between adjacent onsets; HR, Heart rate; Symmetry_RiseDecay, Symmetry (fraction of the period that the cycle is in the rise phase); MCVNN, The median absolute deviation of the NN intervals divided by the median of the NN intervals; PP, Pulse Pressure

The five features most strongly associated with an increased likelihood of shock occurrence are: (1) a higher ECG_HRV_pNN50 (proportion of successive heartbeat intervals differing by more than 50 milliseconds; odds ratio [OR]: 1.01; 95% confidence interval [CI]: 1.01–1.01), (2) a lower RESP_Width_Mean (average time interval between respiratory waveform peaks and troughs; OR: 1.17; 95% CI: 1.06–1.28), (3) a lower RESP_Cycle_Rate_Mean (mean respiratory cycle rate; OR: 0.98; 95% CI: 0.97–0.99), (4) a higher ABP_TimeSBP2DBP_SamEn (sample entropy of the interval between systolic and diastolic peaks; OR: 2.74; 95% CI: 2.24–3.36), and (5) a lower ABP_AmplitudeDBP_Median (median amplitude between consecutive diastolic peaks; OR: 0.95; 95% CI: 0.90–0.99) (Fig. 3; Table 3).

**Table 3:** Simple logistic regression for shock prediction using physiologic waveform features

Variables	OR (95% CI) ^a^	Variables	OR (95% CI) ^a^
ABP_TimeSBP2DBP__SampEn	2.74 (2.24 to 3.36) **	ABP_Psys_Median	0.99 (0.98 to 0.99) **
ECG_HRV_MCVNN	2.47 (1.02 to 5.99) *	ABP_Psys_Max	0.99 (0.99 to 0.99) **
RESP_Cycle_Amplitude_Min	2.07 (1.58 to 2.7) **	ABP_PP_Max	0.99 (0.98 to 0.99) **
RESP_RRV_MFDFA_α1_Peak	1.58 (1.22 to 2.04) **	ABP_Psys_Min	0.98 (0.98 to 0.99) **
RESP_Amplitude_Median	1.41 (1.18 to 1.66) *	ABP_MeanAP_Mean	0.98 (0.98 to 0.98) **
RESP_Cycle_Amplitude_Max	1.18 (1.05 to 1.32) **	RESP_Cycle_Rate_Mean	0.98 (0.97 to 0.99) **
RESP_Width_Mean	1.17 (1.06 to 1.28) **	ABP_AmplitudeDBP__Median	0.95 (0.91 to 0.99) *
ABP_AmplitudeSBP__Median	1.15 (1.11 to 1.2) **	RESP_Cycle_Symmetry_RiseDecay_Max	0.27 (0.14 to 0.54) **
ABP_AmplitudeSBP__Mean	1.13 (1.09 to 1.18) **		
ECG_HRV_pNN_50_	1.01 (1.01 to 1.01) **		
ABP_HR_Mean	1.01 (1.00 to 1.03) **		
RESP_RRV_MeanBB	1.01 (1.01 to 1.02) **		

Multivariable logistic regression models adjusted for age, sex, body mass index, neck and waist circumference. *p < 0.05; **p < 0.01

Abbreviations: ECG, Electrocardiogram; HRV, Heart rate variability; pNN_50_, Proportion of successive heartbeat intervals that differ by more than 50 milliseconds; RESP, Respiratory waveform; ABP, Arterial blood pressure; TimeSBP2DBP, Time interval between systolic peak and subsequent diastolic peak; SampEn, Sample entropy; RRV, Respiratory rate Variability; MFDFA, Multifractal detrended fluctuation analysis; Psys, Systolic blood pressure; MeanBB, The mean value of the breath-to-breath intervals; AmplitudeSBP, amplitudes between consecutive diastolic peaks; MeanAP, Average pressure between adjacent onsets; HR, Heart rate; Symmetry_RiseDecay, Symmetry (fraction of the period that the cycle is in the rise phase); MCVNN, The median absolute deviation of the NN intervals divided by the median of the NN intervals; PP, Pulse Pressure

The density scatter plot of Shapley values in Fig. 3 highlights the top 20 features contributing to the prediction of shock occurrence in the best-performing model (i.e., Weighted Ensemble model). Key features include metrics derived from ABP, RESP, and electrocardiographic heart rate variability (ECG_HRV). BP-derived metrics dominate the list, such as systolic blood pressure (Psys_Median, Psys_Min, Psys_Max), mean arterial pressure (MeanAP_Mean), pulse pressure (PP_Max), systolic amplitude differences (AmplitudeSBP_Mean, AmplitudeSBP_Median), and diastolic amplitude differences (AmplitudeDBP_Median). Respiratory dynamics are represented by RESP_Width_Mean (mean waveform width), RESP_Cycle_Rate_Mean (mean cycle rate), and RESP_Cycle_Amplitude_Max (maximum cycle amplitude). Heart rate variability metrics, including ECG_HRV_pNN50 (proportion of successive heartbeat intervals differing by > 50 ms) and ECG_HRV_MCVNN (median absolute deviation of heartbeat intervals), reflect cardiovascular regulation and autonomic activity.

## Discussion

Shock, associated with high mortality due to an overwhelming systemic immune response, necessitates further development of early identification models to facilitate prompt initiation of targeted therapies. Accordingly, this study developed several machine learning models to predict shock occurrences using physiological signals and related features, while also assessing their importance. The weighted ensemble demonstrated the highest accuracy and AUC across both training/validation and testing datasets. Furthermore, logistic regression models, aimed at confirming the robustness of the documented the top 20 feature importances among selected 65 features, also revealed significant odds ratios between these physiological features and shock occurrence.

Regarding the significance of ECG signal features in predicting shock occurrence, pNN_50_ was identified as the most crucial feature. This feature is validated as an indicator of increased parasympathetic system activity (vagal tone) and overall autonomic balance between the sympathetic and parasympathetic nervous systems. Previous studies have shown that septic shock typically involves complex interactions within the autonomic nervous and immune system [35]. Specifically, the vagus nerve can sense inflammation due to shock, transmitting afferent signals from the periphery to the brainstem. This stimulates efferent vagus nerve activity, leading to the release of acetylcholine in affected organs [36]. Consequently, the inflammatory reflex caused by shock, modulated through vagal control of the immune system, may be reflected by changes in the pNN_50_ measure. In other words, an elevated pNN_50_ may signify increased vagal tone, which could be associated with adverse outcomes in critical conditions related to septic shock. Prior researchers have observed significantly higher pNN_50_ values in septic patients with impaired cerebral autoregulation compared to those with normal function [37]. A related study compared the HRV features derived from a 20-minute Holter test conducted on the first day of enrollment for shock, documenting relatively higher pNN_50_ values in non-survivors compared to survivors [38]. Similarly, another study found that shock patients who were non-survivors without atrial fibrillation demonstrated relatively higher pNN_50_ values compared to survivors, suggesting a comparable parasympathetic tone in both groups [39]. Altogether, the HRV feature, namely pNN_50_, underscored critical alterations in vagal tone during shock, which may reflect severe disturbances in autonomic and immune functions, potentially signaling poorer clinical outcomes in affected patients.

For the parameters related to respiratory waveforms, the average time interval between peaks and troughs and the mean cycle rate were associated with the risk of shock occurrence, serving as predominant predictive features. These relationships may be partly because alterations in respiratory waveform reflect underlying physiological changes related to respiratory effort and cardiac function. Precisely, respiratory waveform variations, including rate, time, and volume, can serve as critical parameters that reflect the dynamic interactions between the respiratory and cardiovascular systems. During shock, the common hemodynamic status is severely compromised, leading to alterations in respiratory patterns or rapid breathing that can serve as indicators of impending cardiovascular collapse [40]. Hence, these detectable changes in respiratory waveform may be able to assess fluid responsiveness, hemodynamic instability and cardiovascular stress. Similar to some previous outcomes, Cannesson et al. demonstrated that respiratory variations in the waveform amplitude of pulse oximetry can predict fluid responsiveness in mechanically ventilated patients, suggesting the possibility of using respiratory waveform variations to assess hemodynamic status [41]. Guo et al. investigated the associations between respiratory rate variations and venous-to-arterial carbon dioxide tension differences in shock patients, demonstrating such patterns may serve as indicators of hemodynamic parameters [42]. Collectively, similar to previous studies, these findings highlight the significance of respiratory waveform variations as critical indicators of hemodynamic instability and may aid in enhancing the prediction and management of shock.

Regarding features related to blood pressure, the sample entropy of the time interval between systolic and subsequent diastolic peaks in ABP ranked as the fourth and fifth most important features. Logistic regression analysis also highlighted significant associations between these parameters and the risk of shock, further evidenced by significant odds ratios. These observations may be attributable to their direct reflection of cardiovascular system functionality and stability. In more details, hemodynamic instability, a common manifestation of shock, presents as irregularities and variability in blood pressure, which are indicative of the sample entropy of ABP intervals [43]. High sample entropy values, indicating variability in ABP intervals, can also be influenced by poor microcirculatory perfusion in organs, a common complication of septic shock [44]. Additionally, the relationship between systolic and diastolic pressures straightforwardly reflects changes in cardiac output and vascular resistance, which are also associated with the severity of shock [45]. Similar to research proposing related indices from ABP, Ospina-Tascón et al. introduced the concept of the diastolic shock index, which is the ratio of HR to diastolic arterial pressure [46]. Their findings indicated that a higher index was associated with increased mortality in shock patients, emphasizing the significance of diastolic pressure and its variability as predictors of shock outcomes. Tang et al. observed that the variability of systolic pressure during the early stage of intensive care unit admission was associated with 28-day mortality in patients with severe sepsis and shock [47]. Taken together, the present findings aligned with previous studies that reveal the importance of ABP variability and hemodynamic parameters in predicting shock outcomes. Considering such dynamic features into predictive models may enhance the early identification and management of shock, potentially improving patient outcomes.

The current study has several strengths. First, this study meticulously crafted approximately 300 features related to ECG, respiratory waveforms, ABP, and SpO_2_ signals as part of comprehensive feature engineering. These were developed to predict the occurrence of shock, thereby enhancing the analytical depth and breadth of the predictive models. Next, by employing mutual information entropy analysis to optimize feature selection, the top 65 features were rigorously selected for further model development. This methodological precision ensures that only the most impactful features are used, optimizing the predictive performance of the models. This study utilized self-sampling techniques, which may more accurately identify features related to the occurrence of shock by considering individual baseline variations. This study also considered the outcomes from previous similar studies that used the same time period but with enhanced feature engineering [48]. Hence, for the established models, the relatively high accuracy achieved highlights the effectiveness of these enhancements. Besides, a detailed feature importance survey was conducted, which provides critical insights into specific physiological markers that are most indicative of shock, aiding clinicians in understanding and potentially anticipating patient needs. To validate the importance of the identified primary features, logistic regression analyses were conducted. These analyses confirmed the robustness and predictive relevance of the selected features, further solidifying their utility in clinical settings.

There are some limitations that need to be considered and addressed in future studies. First, although this study defines shock events using a combination of MAP ≤ 65 mmHg and serum lactate levels ≥ 2 mmol/L, it is recognized that clinically relevant hypotension may occur across a broader MAP range, such as 70–80 mmHg or higher, depending on individual conditions. Besides, lactate is a late biomarker and may not precisely reflect real-time physiological changes prior to shock. This limitation may affect the sensitivity and specificity of the predictive model. Therefore, the definition used in this study may not capture all relevant hypotensive events, potentially limiting the clinical sensitivity and generalizability of the model. Future studies should consider incorporate more nuanced or context-specific criteria for hypotension to better reflect the real intensive care patient population. Second, this study employed feature engineering techniques for four physiological signals to incorporate a diverse range of input features, aiming to enhance robustness. However, due to the inclusion criteria and inherent missing data, the number of eligible patient cases may have been limited, potentially leading to a higher prevalence of shock events compared to the general ICU population. Besides, due to the retrospective design, normal (non-shock) data were labelled either from post-shock periods up to seven days later or from pre-shock periods (24-hour before). This variability in data labelling may have partially influenced model performance and limits the generalizability of the research findings. Additionally, since the data were retrieved using a self-controlled approach, which may have resulted in a higher prevalence of shock events, the model performance metrics—particularly those sensitive to class imbalance, such as positive predictive value—were not further evaluated, as they may not fully represent the robustness of the established models across diverse clinical settings. Future studies should consider more standardized conditions for labelling normal data to enhance model reliability and applicability. Regarding ECG data, although a bandpass filter was applied and heart peaks were interpolated in instances where the recorded HR exceeded a predefined threshold, the inherent limitations associated with the peak-picking resampling method in the MIMIC dataset may still have introduced distortions in the RRI series and affected the accuracy of HRV-related features. More precisely, utilizing heart peak interpolation may only mitigated the effects of extreme values, which did not fully account for potential distortions across the entire HR spectrum. Future study should incorporate data quality assessments or alternative signal preprocessing techniques to better ensure the robustness of HRV analysis. Next, the timeframe length used to incorporate physiological data for shock prediction was limited to a 30-minute segment based on common clinical practices, which might not be optimal for all clinical cases. Moreover, this study employed mutual information-based feature selection to identify the top 65 features for optimal shock prediction performance; however, this approach has inherent limitations. The selection process may not fully capture the dynamic interactions between physiological parameters and does not inherently account for feature redundancy or collinearity, which could affect model interpretability and generalizability. Future work may consider exploring the impact of different time window lengths on model performance, as well as comparing different combinations of features to further optimize predictive accuracy and robustness. Additionally, while this research utilized a public database, further studies are needed to adapt the models to hardware with varying data sampling rates to ensure broader applicability and accuracy across different clinical settings.

## Conclusion

This study demonstrated the feasibility of predicting shock one hour before its onset using four continuously monitored physiological waveforms: ABP, ECG, respiratory waveforms, and SpO_2_. By incorporating feature engineering grounded in physiological concepts and leveraging self-sampling data, the developed model achieved a high AUC, highlighting its potential for early and accurate shock prediction. The use of self-controlled data allowed for the improvement in predictive performance, ensuring that predictions were more clinically meaningful while maintaining high sensitivity. Furthermore, mutual information-based feature selection helped identify the most relevant physiological markers, improving model interpretability and robustness. The integration of a weighted ensemble approach further optimized prediction performance by balancing sensitivity and specificity. These findings reinforce the potential of machine learning-driven physiological waveform analysis as a non-invasive, early warning system for shock prediction, supporting proactive ICU monitoring and timely intervention. Given that continuous ABP monitoring is often limited in clinical practice, future study should explore more widely accessible data to improve applicability and generalizability.

## Electronic supplementary material

Below is the link to the electronic supplementary material.

Supplementary Material 1

## Data Availability

The dataset utilized in this study is derived from the Medical Information Mart for Intensive Care (MIMIC-III), a comprehensive database from a single center that records detailed patient information from critical care units in a large tertiary hospital. Due to the inclusion of sensitive patient data, the datasets are not openly accessible to the public. For further details about MIMIC-III, please visit their official website (https://mimic.mit.edu/about/mimic/). Researchers interested in accessing this data must complete the CITI course titled “Data or Specimens Only Research” (https://www.citiprogram.org/index.cfm?pageID=154%26icat=0%26ac=0) and apply for credentialed access via PhysioNet (https://physionet.org/content/mimiciii/).
