# Supplementary material for: Advancing shock prediction: leveraging prior knowledge and self-controlled data for enhanced model accuracy and generalizability
Source: BMC Med Inform Decis Mak. 2025 Jul 14;25:262. doi: 10.1186/s12911-025-03108-2 (PMC12261771; doi:10.1186/s12911-025-03108-2)
Supplement: Supplementary file 1 — Supplementary Material 1 [file 12911_2025_3108_MOESM1_ESM.docx]

**Supplementary Table 1. Comparison of Simple Logistic Regression Outcomes for Blood Pressure data Between In-Shock and Normal Periods**

| **Variables** | **OR (95% CI) ^a^** | **Variables** | **OR (95% CI) ^a^** |
| --- | --- | --- | --- |
| ABP_AmplitudeSBP__Lyapunov | 10.56 (0.32 to 347.74) | **ABP_HR_Median** | **0.99 (0.99 to 1.01) ^**^** |
| **ABP_AmplitudeDBP__Lyapunov** | **3.79 (2.82 to 4.87) ^*^** | **ABP_Pdias_Max** | **0.99 (0.99 to 0.99) ^**^** |
| ABP_AmplitudeSBP__Min | 3.78 (0.61 to 23.56) | **ABP_Psys_Max** | **0.99 (0.99 to 0.99) ^**^** |
| ABP_TimeSBP2DBP__STD | 3.11 (0.22 to 44.2) | **ABP_Psys_Kurtosis** | **0.99 (0.98 to 1) ^*^** |
| ABP_TimeSBP2DBP__Mean | 2.96 (0.68 to 12.83) | **ABP_HR_Min** | **0.99 (0.98 to 0.99) ^**^** |
| **ABP_TimeSBP2DBP__Lyapunov** | **2.79 (1.08 to 7.21) ^*^** | **ABP_MAP_Max** | **0.99 (0.98 to 0.99) ^**^** |
| **ABP_TimeSBP2DBP__SampEn** | **2.74 (2.24 to 3.36) ^**^** | **ABP_MeanAP_Max** | **0.99 (0.98 to 0.99) ^**^** |
| **ABP_AmplitudeDBP__Hurst** | **2.37 (1.41 to 4.01) ^**^** | **ABP_PP_Kurtosis** | **0.99 (0.98 to 0.99) ^**^** |
| **ABP_MeanAP_Hurst** | **1.59 (1.05 to 2.41) ^*^** | **ABP_PP_Mean** | **0.99 (0.98 to 0.99) ^**^** |
| ABP_MAP_Hurst | 1.47 (0.96 to 2.26) | **ABP_PP_Median** | **0.99 (0.98 to 0.99) ^**^** |
| ABP_Psys_Hurst | 1.46 (0.95 to 2.26) | **ABP_PP_Min** | **0.99 (0.98 to 0.99) ^**^** |
| ABP_AmplitudeDBP__Min | 1.45 (0.98 to 1.92) | **ABP_Psys_Mean** | **0.99 (0.98 to 0.99) ^**^** |
| ABP_TimeSBP2DBP__Median | 1.43 (0.37 to 5.58) | **ABP_Psys_Median** | **0.99 (0.98 to 0.99) ^**^** |
| ABP_TimeSBP2DBP__Hurst | 1.31 (0.84 to 2.01) | **ABP_PP_Max** | **0.99 (0.98 to 0.99) ^**^** |
| **ABP_MeanAP_SampEn** | **1.24 (1.05 to 1.45) ^*^** | ABP_MAP_Kurtosis | 0.99 (0.97 to 1.01) |
| **ABP_AmplitudeSBP__SampEn** | **1.19 (1.02 to 1.41) ^*^** | ABP_PP_SampEn | 0.99 (0.86 to 1.14) |
| **ABP_AmplitudeSBP__Median** | **1.15 (1.11 to 1.21) ^**^** | ABP_AmplitudeSBP__Hurst | 0.99 (0.61 to 1.59) |
| **ABP_AmplitudeSBP__Mean** | **1.13 (1.09 to 1.18) ^**^** | **ABP_Pdias_Mean** | **0.98 (0.98 to 0.99) ^**^** |
| **ABP_AmplitudeSBP__STD** | **1.13 (1.08 to 1.19) ^**^** | **ABP_Pdias_Median** | **0.98 (0.98 to 0.99) ^**^** |
| ABP_Pdias_Hurst | 1.13 (0.75 to 1.71) | **ABP_Psys_Min** | **0.98 (0.98 to 0.99) ^**^** |
| ABP_AmplitudeDBP__SampEn | 1.12 (0.96 to 1.31) | **ABP_MeanAP_Mean** | **0.98 (0.98 to 0.98) ^**^** |
| **ABP_Psys_Skewness** | **1.11 (1.03 to 1.17) ^**^** | **ABP_MeanAP_Median** | **0.98 (0.98 to 0.98) ^**^** |
| ABP_PP_Hurst | 1.09 (0.72 to 1.66) | **ABP_HR_Skewness** | **0.98 (0.97 to 1.01) ^*^** |
| ABP_Psys_SampEn | 1.08 (0.93 to 1.26) | **ABP_MeanAP_Kurtosis** | **0.98 (0.97 to 0.99) ^**^** |
| **ABP_PP_Skewness** | **1.06 (1.01 to 1.11) ^*^** | **ABP_Pdias_Min** | **0.98 (0.97 to 0.99) ^**^** |
| **ABP_PP_STD** | **1.04 (1.02 to 1.06) ^**^** | **ABP_MAP_Mean** | **0.98 (0.97 to 0.98) ^**^** |
| **ABP_Psys_STD** | **1.03 (1.02 to 1.05) ^**^** | **ABP_MAP_Median** | **0.98 (0.97 to 0.98) ^**^** |
| **ABP_HR_STD** | **1.02 (1.01 to 1.03) ^**^** | **ABP_MAP_Min** | **0.98 (0.97 to 0.98) ^**^** |
| **ABP_TimeSBP2DBP__Kurtosis** | **1.02 (1.01 to 1.03) ^**^** | **ABP_MeanAP_Min** | **0.98 (0.97 to 0.98) ^**^** |
| **ABP_AmplitudeSBP__Max** | **1.02 (1.01 to 1.03) ^*^** | ABP_Pdias_STD | 0.98 (0.95 to 1.01) |
| ABP_AmplitudeDBP__Kurtosis | 1.02 (1.01 to 1.03) | ABP_MAP_SampEn | 0.97 (0.82 to 1.15) |
| ABP_MAP_STD | 1.01 (0.99 to 1.04) | **ABP_TimeSBP2DBP__Skewness** | **0.96 (0.94 to 0.97) ^**^** |
| **ABP_AmplitudeSBP__Kurtosis** | **1.01 (0.99 to 1.03) ^*^** | ABP_AmplitudeDBP__Mean | 0.96 (0.91 to 1.01) |
| **ABP_HR_Mean** | **1.01 (1.00 to 1.03) ^*^** | ABP_AmplitudeDBP__Median | 0.95 (0.91 to 0.99) |
| ABP_HR_Kurtosis | 1.01 (0.99 to 1.03) | **ABP_AmplitudeSBP__Skewness** | **0.94 (0.91 to 0.97) ^**^** |
| ABP_HR_Max | 1.01 (0.99 to 1.03) | **ABP_AmplitudeDBP__STD** | **0.94 (0.89 to 1.01) ^*^** |
| ABP_AmplitudeDBP__Max | 1.01 (0.99 to 1.02) | **ABP_MeanAP_Skewness** | **0.91 (0.84 to 0.97) ^**^** |
| ABP_Pdias_Kurtosis | 1.01 (0.99 to 1.02) | ABP_TimeSBP2DBP__Min | 0.91 (0.11 to 7.36) |
| ABP_AmplitudeDBP__Skewness | 1.01 (0.98 to 1.04) | **ABP_HR_SampEn** | **0.85 (0.75 to 0.97) ^*^** |
| ABP_MeanAP_STD | 1.01 (0.98 to 1.03) | ABP_TimeSBP2DBP__Max | 0.65 (0.4 to 1.05) |
| ABP_Pdias_Skewness | 1.01 (0.95 to 1.06) | **ABP_HR_Hurst** | **0.63 (0.44 to 0.88) ^*^** |
| ABP_MAP_Skewness | 1.01 (0.92 to 1.09) | **ABP_Pdias_Lyapunov** | **0.41 (0.01 to 0.81) ^*^** |
| ABP_Pdias_SampEn | 1.01 (0.86 to 1.16) | **ABP_Psys_Lyapunov** | **0.25 (0.01 to 0.54) ^*^** |
|  |  | ABP_PP_Lyapunov | 0.25 (0 to 51.15) |
|  |  | **ABP_MAP_Lyapunov** | **0.11 (0.01 to 0.25) ^*^** |
|  |  | **ABP_MeanAP_Lyapunov** | **0.11 (0.01 to 0.24) ^**^** |
|  |  | **ABP_HR_Lyapunov** | **0.05 (0.02 to 0.16) ^**^** |
| Multivariable logistic regression models adjusted for age, sex, body mass index, neck and waist circumference.  **p* < 0.05; ***p* < 0.01. | | | |

| ***Definition of abbreviations:*** | | | |
| --- | --- | --- | --- |
| ***Feature Code*** | ***Feature Description*** | ***Metric*** | ***Metric Description*** |
| *ABP_Psys_Min* | *Systolic BP (Ps)* | *Min* | *Minimum value* |
| *ABP_Psys_Max* | *Systolic BP (Ps)* | *Max* | *Maximum value* |
| *ABP_Psys_Mean* | *Systolic BP (Ps)* | *Mean* | *Average value* |
| *ABP_Psys_Median* | *Systolic BP (Ps)* | *Median* | *Median value* |
| *ABP_Psys_STD* | *Systolic BP (Ps)* | *STD* | *Standard Deviation* |
| *ABP_Psys_Skewness* | *Systolic BP (Ps)* | *Skewness* | *Skewness* |
| *ABP_Psys_Kurtosis* | *Systolic BP (Ps)* | *Kurtosis* | *Kurtosis* |
| *ABP_Psys_Hurst* | *Systolic BP (Ps)* | *Hurst* | *Hurst exponent* |
| *ABP_Psys_Lyapunov* | *Systolic BP (Ps)* | *Lyapunov* | *Lyapunov exponent* |
| *ABP_Psys_SampEn* | *Systolic BP (Ps)* | *SampEn* | *Sample Entropy* |
| *ABP_Pdias_Min* | *Diastolic BP (Pd)* | *Min* | *Minimum value* |
| *ABP_Pdias_Max* | *Diastolic BP (Pd)* | *Max* | *Maximum value* |
| *ABP_Pdias_Mean* | *Diastolic BP (Pd)* | *Mean* | *Average value* |
| *ABP_Pdias_Median* | *Diastolic BP (Pd)* | *Median* | *Median value* |
| *ABP_Pdias_STD* | *Diastolic BP (Pd)* | *STD* | *Standard Deviation* |
| *ABP_Pdias_Skewness* | *Diastolic BP (Pd)* | *Skewness* | *Skewness* |
| *ABP_Pdias_Kurtosis* | *Diastolic BP (Pd)* | *Kurtosis* | *Kurtosis* |
| *ABP_Pdias_Hurst* | *Diastolic BP (Pd)* | *Hurst* | *Hurst exponent* |
| *ABP_Pdias_Lyapunov* | *Diastolic BP (Pd)* | *Lyapunov* | *Lyapunov exponent* |
| *ABP_Pdias_SampEn* | *Diastolic BP (Pd)* | *SampEn* | *Sample Entropy* |
| *ABP_PP_Min* | *Pulse Pressure (Ps - Pd)* | *Min* | *Minimum value* |
| *ABP_PP_Max* | *Pulse Pressure (Ps - Pd)* | *Max* | *Maximum value* |
| *ABP_PP_Mean* | *Pulse Pressure (Ps - Pd)* | *Mean* | *Average value* |
| *ABP_PP_Median* | *Pulse Pressure (Ps - Pd)* | *Median* | *Median value* |
| *ABP_PP_STD* | *Pulse Pressure (Ps - Pd)* | *STD* | *Standard Deviation* |
| *ABP_PP_Skewness* | *Pulse Pressure (Ps - Pd)* | *Skewness* | *Skewness* |
| *ABP_PP_Kurtosis* | *Pulse Pressure (Ps - Pd)* | *Kurtosis* | *Kurtosis* |
| *ABP_PP_Hurst* | *Pulse Pressure (Ps - Pd)* | *Hurst* | *Hurst exponent* |
| *ABP_PP_Lyapunov* | *Pulse Pressure (Ps - Pd)* | *Lyapunov* | *Lyapunov exponent* |
| *ABP_PP_SampEn* | *Pulse Pressure (Ps - Pd)* | *SampEn* | *Sample Entropy* |
| *ABP_MeanAP_Min* | *Mean Pressure (Pm) : average pressure between adjacent onsets.* | *Min* | *Minimum value* |
| *ABP_MeanAP_Max* | *Mean Pressure (Pm) : average pressure between adjacent onsets.* | *Max* | *Maximum value* |
| *ABP_MeanAP_Mean* | *Mean Pressure (Pm) : average pressure between adjacent onsets.* | *Mean* | *Average value* |
| *ABP_MeanAP_Median* | *Mean Pressure (Pm) : average pressure between adjacent onsets.* | *Median* | *Median value* |
| *ABP_MeanAP_STD* | *Mean Pressure (Pm) : average pressure between adjacent onsets.* | *STD* | *Standard Deviation* |
| *ABP_MeanAP_Skewness* | *Mean Pressure (Pm) : average pressure between adjacent onsets.* | *Skewness* | *Skewness* |
| *ABP_MeanAP_Kurtosis* | *Mean Pressure (Pm) : average pressure between adjacent onsets.* | *Kurtosis* | *Kurtosis* |
| *ABP_MeanAP_Hurst* | *Mean Pressure (Pm) : average pressure between adjacent onsets.* | *Hurst* | *Hurst exponent* |
| *ABP_MeanAP_Lyapunov* | *Mean Pressure (Pm) : average pressure between adjacent onsets.* | *Lyapunov* | *Lyapunov exponent* |
| *ABP_MeanAP_SampEn* | *Mean Pressure (Pm) : average pressure between adjacent onsets.* | *SampEn* | *Sample Entropy* |
| *ABP_HR_Min* | *Heart rate (60/T) (HR)* | *Min* | *Minimum value* |
| *ABP_HR_Max* | *Heart rate (60/T) (HR)* | *Max* | *Maximum value* |
| *ABP_HR_Mean* | *Heart rate (60/T) (HR)* | *Mean* | *Average value* |
| *ABP_HR_Median* | *Heart rate (60/T) (HR)* | *Median* | *Median value* |
| *ABP_HR_STD* | *Heart rate (60/T) (HR)* | *STD* | *Standard Deviation* |
| *ABP_HR_Skewness* | *Heart rate (60/T) (HR)* | *Skewness* | *Skewness* |
| *ABP_HR_Kurtosis* | *Heart rate (60/T) (HR)* | *Kurtosis* | *Kurtosis* |
| *ABP_HR_Hurst* | *Heart rate (60/T) (HR)* | *Hurst* | *Hurst exponent* |
| *ABP_HR_Lyapunov* | *Heart rate (60/T) (HR)* | *Lyapunov* | *Lyapunov exponent* |
| *ABP_HR_SampEn* | *Heart rate (60/T) (HR)* | *SampEn* | *Sample Entropy* |
| *ABP_MAP_Min* | *MAP (Mean Arterial Pressure)* | *Min* | *Minimum value* |
| *ABP_MAP_Max* | *MAP (Mean Arterial Pressure)* | *Max* | *Maximum value* |
| *ABP_MAP_Mean* | *MAP (Mean Arterial Pressure)* | *Mean* | *Average value* |
| *ABP_MAP_Median* | *MAP (Mean Arterial Pressure)* | *Median* | *Median value* |
| *ABP_MAP_STD* | *MAP (Mean Arterial Pressure)* | *STD* | *Standard Deviation* |
| *ABP_MAP_Skewness* | *MAP (Mean Arterial Pressure)* | *Skewness* | *Skewness* |
| *ABP_MAP_Kurtosis* | *MAP (Mean Arterial Pressure)* | *Kurtosis* | *Kurtosis* |
| *ABP_MAP_Hurst* | *MAP (Mean Arterial Pressure)* | *Hurst* | *Hurst exponent* |
| *ABP_MAP_Lyapunov* | *MAP (Mean Arterial Pressure)* | *Lyapunov* | *Lyapunov exponent* |
| *ABP_MAP_SampEn* | *MAP (Mean Arterial Pressure)* | *SampEn* | *Sample Entropy* |
| *ABP_TimeSBP2DBP_Min* | *Time interval between systolic peak and subsequent diastolic peak* | *Min* | *Minimum value* |
| *ABP_TimeSBP2DBP_Max* | *Time interval between systolic peak and subsequent diastolic peak* | *Max* | *Maximum value* |
| *ABP_TimeSBP2DBP_Mean* | *Time interval between systolic peak and subsequent diastolic peak* | *Mean* | *Average value* |
| *ABP_TimeSBP2DBP_Median* | *Time interval between systolic peak and subsequent diastolic peak* | *Median* | *Median value* |
| *ABP_TimeSBP2DBP_STD* | *Time interval between systolic peak and subsequent diastolic peak* | *STD* | *Standard Deviation* |
| *ABP_TimeSBP2DBP_Skewness* | *Time interval between systolic peak and subsequent diastolic peak* | *Skewness* | *Skewness* |
| *ABP_TimeSBP2DBP_Kurtosis* | *Time interval between systolic peak and subsequent diastolic peak* | *Kurtosis* | *Kurtosis* |
| *ABP_TimeSBP2DBP_Hurst* | *Time interval between systolic peak and subsequent diastolic peak* | *Hurst* | *Hurst exponent* |
| *ABP_TimeSBP2DBP_Lyapunov* | *Time interval between systolic peak and subsequent diastolic peak* | *Lyapunov* | *Lyapunov exponent* |
| *ABP_TimeSBP2DBP_SampEn* | *Time interval between systolic peak and subsequent diastolic peak* | *SampEn* | *Sample Entropy* |
| *ABP_AmplitudeSBP_Min* | *Amplitudes between consecutive systolic peaks* | *Min* | *Minimum value* |
| *ABP_AmplitudeSBP_Max* | *Amplitudes between consecutive systolic peaks* | *Max* | *Maximum value* |
| *ABP_AmplitudeSBP_Mean* | *Amplitudes between consecutive systolic peaks* | *Mean* | *Average value* |
| *ABP_AmplitudeSBP_Median* | *Amplitudes between consecutive systolic peaks* | *Median* | *Median value* |
| *ABP_AmplitudeSBP_STD* | *Amplitudes between consecutive systolic peaks* | *STD* | *Standard Deviation* |
| *ABP_AmplitudeSBP_Skewness* | *Amplitudes between consecutive systolic peaks* | *Skewness* | *Skewness* |
| *ABP_AmplitudeSBP_Kurtosis* | *Amplitudes between consecutive systolic peaks* | *Kurtosis* | *Kurtosis* |
| *ABP_AmplitudeSBP_Hurst* | *Amplitudes between consecutive systolic peaks* | *Hurst* | *Hurst exponent* |
| *ABP_AmplitudeSBP_Lyapunov* | *Amplitudes between consecutive systolic peaks* | *Lyapunov* | *Lyapunov exponent* |
| *ABP_AmplitudeSBP_SampEn* | *Amplitudes between consecutive systolic peaks* | *SampEn* | *Sample Entropy* |
| *ABP_AmplitudeDBP_Min* | *Amplitudes between consecutive diastolic peaks* | *Min* | *Minimum value* |
| *ABP_AmplitudeDBP_Max* | *Amplitudes between consecutive diastolic peaks* | *Max* | *Maximum value* |
| *ABP_AmplitudeDBP_Mean* | *Amplitudes between consecutive diastolic peaks* | *Mean* | *Average value* |
| *ABP_AmplitudeDBP_Median* | *Amplitudes between consecutive diastolic peaks* | *Median* | *Median value* |
| *ABP_AmplitudeDBP_STD* | *Amplitudes between consecutive diastolic peaks* | *STD* | *Standard Deviation* |
| *ABP_AmplitudeDBP_Skewness* | *Amplitudes between consecutive diastolic peaks* | *Skewness* | *Skewness* |
| *ABP_AmplitudeDBP_Kurtosis* | *Amplitudes between consecutive diastolic peaks* | *Kurtosis* | *Kurtosis* |
| *ABP_AmplitudeDBP_Hurst* | *Amplitudes between consecutive diastolic peaks* | *Hurst* | *Hurst exponent* |
| *ABP_AmplitudeDBP_Lyapunov* | *Amplitudes between consecutive diastolic peaks* | *Lyapunov* | *Lyapunov exponent* |
| *ABP_AmplitudeDBP_SampEn* | *Amplitudes between consecutive diastolic peaks* | *SampEn* | *Sample Entropy* |

**Supplementary Table 2. Comparison of Simple Logistic Regression Outcomes for Electrocardiogram Data Between In-Shock and Normal Periods**

| **Variables** | **OR (95% CI) ^a^** | **Variables** | **OR (95% CI) ^a^** |
| --- | --- | --- | --- |
| ECG_HRV_LF | 31.36 (0.53 to 1867.05) | ECG_HRV_LFHF | 0.99 (0.99 to 1) |
| ECG_HRV_HF | 13.44 (1.59 to 113.25) ^*^ | ECG_HRV_GI | 0.99 (0.91 to 1.09) |
| ECG_HRV_HFn | 2.89 (2.04 to 4.11) ^**^ | ECG_HRV_SI | 0.99 (0.9 to 1.08) |
| ECG_HRV_MFDFA_α1_Increment | 2.88 (2.14 to 3.89) ^**^ | ECG_HRV_ApEn | 0.99 (0.88 to 1.12) |
| ECG_HRV_MCVNN | 2.47 (1.02 to 5.99) | ECG_HRV_SampEn | 0.98 (0.87 to 1.11) |
| ECG_HRV_HFD | 2.03 (1.27 to 3.27) ^**^ | ECG_HRV_CD | 0.98 (0.86 to 1.12) |
| ECG_HRV_C1d | 1.79 (0.69 to 4.62) | ECG_HRV_CSI | 0.97 (0.93 to 1.01) |
| ECG_HRV_Ca | 1.48 (0.25 to 8.81) | ECG_HRV_RCMSEn | 0.96 (0.86 to 1.08) |
| ECG_HRV_SD1SD2 | 1.43 (1.23 to 1.68) ^**^ | ECG_HRV_MFDFA_α2_Fluctuation | 0.96 (0.42 to 2.2) |
| ECG_HRV_MFDFA_α1_Asymmetry | 1.42 (1.17 to 1.72) ^**^ | ECG_HRV_CMSEn | 0.95 (0.79 to 1.15) |
| ECG_HRV_LZC | 1.35 (1.02 to 1.79) ^*^ | ECG_HRV_MFDFA_α1_Delta | 0.93 (0.88 to 0.97) ^**^ |
| ECG_HRV_VHF | 1.31 (1.27 to1.36) ^**^ | ECG_HRV_MFDFA_α2_Delta | 0.91 (0.83 to 0.98) ^*^ |
| ECG_HRV_MFDFA_α1_Width | 1.27 (1.16 to 1.4) ^**^ | ECG_HRV_MFDFA_α1_Peak | 0.9 (0.76 to 1.06) |
| ECG_HRV_C2a | 1.27 (0.75 to 2.16) | ECG_HRV_MFDFA_α1_Max | 0.89 (0.85 to 0.94) ^**^ |
| ECG_HRV_CVNN | 1.25 (0.9 to 1.75) | ECG_HRV_DFA_α1 | 0.88 (0.71 to 1.1) |
| ECG_HRV_LFn | 1.24 (0.74 to 2.08) | ECG_HRV_C2d | 0.79 (0.46 to 1.34) |
| ECG_HRV_CVI | 1.23 (1.15 to 1.31) ^**^ | ECG_HRV_PSS | 0.71 (0.22 to 2.25) |
| ECG_HRV_CVSD | 1.2 (0.94 to 1.53) | ECG_HRV_Cd | 0.68 (0.11 to 4.03) |
| ECG_HRV_MFDFA_α2_Asymmetry | 1.17 (0.97 to 1.41) | ECG_HRV_MFDFA_α2_Max | 0.64 (0.56 to 0.74) ^**^ |
| ECG_HRV_MFDFA_α2_Width | 1.15 (1 to 1.32) | ECG_HRV_MFDFA_α2_Peak | 0.61 (0.51 to 0.72) ^**^ |
| ECG_HRV_MFDFA_α1_Mean | 1.15 (1 to 1.31) | ECG_HRV_MFDFA_α2_Mean | 0.61 (0.5 to 0.74) ^**^ |
| ECG_HRV_ShanEn | 1.11 (1.04 to 1.15) ^**^ | ECG_HRV_PIP | 0.59 (0.25 to 1.37) |
| ECG_HRV_MFDFA_α1_Fluctuation | 1.09 (1.01 to 1.17) ^**^ | ECG_HRV_C1a | 0.56 (0.22 to 1.45) |
| ECG_HRV_MFDFA_α2_Increment | 1.09 (0.81 to 1.47) | ECG_HRV_MSEn | 0.55 (0.47 to 0.64) ^**^ |
| ECG_HRV_FuzzyEn | 1.07 (0.9 to 1.28) | ECG_HRV_DFA_α2 | 0.53 (0.44 to 0.64) ^**^ |
| ECG_HRV_LnHF | 1.06 (1.03 to 1.1) ^**^ | ECG_HRV_IALS | 0.42 (0.25 to 0.71) ^**^ |
| ECG_HRV_KFD | 1.03 (1 to 1.05) | ECG_HRV_PAS | 0.41 (0.27 to 0.63) ^**^ |
| ECG_HRV_RMSSD | 1.02 (0.99 to 1.05) | ECG_HRV_VLF | 0.41 (0.01 to 0.83) |
| ECG_HRV_SD1a | 1.02 (0.99 to 1.05) | ECG_HRV_SDNNI5 | 0.02 (0.01 to 0.03) ^**^ |
| ECG_HRV_MedianNN | 1.02 (0.99 to 1.04) ^**^ | ECG_HRV_ULF | 0.02 (0.01 to 0.03) ^**^ |
| ECG_HRV_Prc80NN | 1.02 (0.99 to 1.04) ^**^ | ECG_HRV_SDANN5 | 0.01 (0.01 to 0.02) ^**^ |
| ECG_HRV_HTI | 1.02 (0.99 to 1.04) |  |  |
| ECG_HRV_MinNN | 1.02 (0.99 to 1.04) |  |  |
| ECG_HRV_SD2a | 1.02 (0.99 to 1.04) |  |  |
| ECG_HRV_SDNNd | 1.02 (0.99 to 1.04) |  |  |
| ECG_HRV_SDNNI1 | 1.02 (0.99 to 1.04) |  |  |
| ECG_HRV_SDNNI2 | 1.02 (0.99 to 1.04) |  |  |
| ECG_HRV_SDANN1 | 1.02 (0.98 to 1.06) |  |  |
| ECG_HRV_SDANN2 | 1.02 (0.98 to 1.06) |  |  |
| ECG_HRV_pNN_20_ | 1.01 (1.01 to 1.01) ^**^ |  |  |
| ECG_HRV_pNN_50_ | 1.01 (1.01 to 1.01) ^**^ |  |  |
| ECG_HRV_IQRNN | 1.01 (0.99 to 1.03) ^**^ |  |  |
| ECG_HRV_MeanNN | 1.01 (0.99 to 1.03) ^*^ |  |  |
| ECG_HRV_CSI_Modified | 1.01 (0.99 to 1.03) |  |  |
| ECG_HRV_MadNN | 1.01 (0.99 to 1.03) |  |  |
| ECG_HRV_MaxNN | 1.01 (0.99 to 1.03) |  |  |
| ECG_HRV_PI | 1.01 (0.99 to 1.03) |  |  |
| ECG_HRV_Prc20NN | 1.01 (0.99 to 1.03) |  |  |
| ECG_HRV_S | 1.01 (0.99 to 1.03) |  |  |
| ECG_HRV_SD1 | 1.01 (0.99 to 1.03) |  |  |
| ECG_HRV_SD1d | 1.01 (0.99 to 1.03) |  |  |
| ECG_HRV_SD2 | 1.01 (0.99 to 1.03) |  |  |
| ECG_HRV_SD2d | 1.01 (0.99 to 1.03) |  |  |
| ECG_HRV_SDNN | 1.01 (0.99 to 1.03) |  |  |
| ECG_HRV_SDNNa | 1.01 (0.99 to 1.03) |  |  |
| ECG_HRV_SDSD | 1.01 (0.99 to 1.03) |  |  |
| ECG_HRV_TINN | 1.01 (0.99 to 1.03) |  |  |
| ECG_HRV_AI | 1.01 (0.93 to 1.07) |  |  |
| Multivariable logistic regression models adjusted for age, sex, body mass index, neck and waist circumference.  **p* < 0.05; ***p* < 0.01. | | | |

| ***Definition of abbreviations:*** | |
| --- | --- |
| ***Feature*** | ***Description*** |
| *ECG_HRV_MeanNN* | *Mean of the normal beat intervals (denoted NN intervals)* |
| *ECG_HRV_SDNN* | *Standard deviation of the NN intervals over the entire recording.* |
| *ECG_HRV_SDANN1* | *Standard deviation of average NN for each 1 min segment of the HR time series.* |
| *ECG_HRV_SDNNI1* | *Mean of the standard deviations of NN calculated per 1 min segment.* |
| *ECG_HRV_SDANN2* | *Similar to SDANN1 but for 2 min segment* |
| *ECG_HRV_SDNNI2* | *Similar to SDNNI1 but for 2 min segment* |
| *ECG_HRV_SDANN5* | *Similar to SDANN1 but for 5 min segment* |
| *ECG_HRV_SDNNI5* | *Similar to SDNNI1 but for 5 min segment* |
| *ECG_HRV_RMSSD* | *Root mean square of successive difference of NN intervals, indicative of short-term variations in HR.* |
| *ECG_HRV_SDSD* | *Standard deviation of the successive difference of NN intervals, indicative of short-term variations in HR.* |
| *ECG_HRV_CVNN* | *Coefficient of Variation of NN, ratio of the standard deviation to the mean.* |
| *ECG_HRV_CVSD* | *The root mean square of successive differences (RMSSD) divided by the mean of the NN intervals (MeanNN).* |
| *ECG_HRV_MedianNN* | *Median value of the NN intervals.* |
| *ECG_HRV_MadNN* | *Median Absolute Deviation of NN intervals, a median-based index of variability.* |
| *ECG_HRV_MCVNN* | *The median absolute deviation of the NN intervals (MadNN) divided by the median of the NN intervals (MedianNN).* |
| *ECG_HRV_IQRNN* | *Interquartile range of the NN intervals.* |
| *ECG_HRV_Prc20NN* | *20th percentile of the NN intervals.* |
| *ECG_HRV_Prc80NN* | *80th percentile of the NN intervals.* |
| *ECG_HRV_pNN50* | *Proportion of successive NN intervals that are larger than 50 ms.* |
| *ECG_HRV_pNN20* | *Proportion of successive NN intervals that are larger than 20 ms.* |
| *ECG_HRV_MinNN* | *Minimum value of the NN intervals.* |
| *ECG_HRV_MaxNN* | *Maximum value of the NN intervals.* |
| *ECG_HRV_HTI* | *HRV triangular index, derived from geometric representation of NN.* |
| *ECG_HRV_TINN* | *Triangular interpolation of NN histogram, derived from geometric representation of NN.* |
| *ECG_HRV_ULF* | *Ultra-low frequency component of the HRV power spectrum (≤0.003 Hz).* |
| *ECG_HRV_VLF* | *Very low frequency component of the HRV power spectrum (0.0033–0.04 Hz).* |
| *ECG_HRV_LF* | *Low frequency component of the HRV power spectrum (0.04–0.15 Hz).* |
| *ECG_HRV_HF* | *High frequency component of the HRV power spectrum (0.15–0.4 Hz), indicative of parasympathetic modulation.* |
| *ECG_HRV_VHF* | *Very high frequency component of HRV (0.4 - 0.5 Hz)* |
| *ECG_HRV_LFHF* | *Ratio of low frequency to high frequency power, a measure of the sympathovagal balance.* |
| *ECG_HRV_LFn* | *Normalized LF, less varied by methodological differences and changes in total power.* |
| *ECG_HRV_HFn* | *Normalized HF, less varied by methodological differences and changes in total power.* |
| *ECG_HRV_LnHF* | *Natural logarithm of HF, an estimator of vagal tone.* |
| *ECG_HRV_SD1* | *Standard deviation of points perpendicular to the identity line in Poincaré plot, equivalent to RMSSD.* |
| *ECG_HRV_SD2* | *Standard deviation of points parallel to the line of identity in Poincaré plot, equivalent to SDNN.* |
| *ECG_HRV_SD1SD2* | *Ratio of SD1 to SD2, reflecting sympathovagal balance in Poincaré plot.* |
| *ECG_HRV_S* | *Area of ellipse described by SD1 and SD2 (pi * SD1 * SD2). It is proportional to  SD1SD2.* |
| *ECG_HRV_CSI* | *The Cardiac Sympathetic Index is a measure of cardiac sympathetic function independent of vagal activity, calculated by dividing the longitudinal variability of the Poincaré plot (4*SD2) by its transverse variability (4*SD1).* |
| *ECG_HRV_CVI* | *The Cardiac Vagal Index is an index of cardiac parasympathetic function (vagal activity unaffected by sympathetic activity), and is equal equal to the logarithm of the product of longitudinal (4*SD2) and transverse variability (4*SD1).* |
| *ECG_HRV_CSI_Modified* | *Modified version of the CSI, obtained by dividing the square of the longitudinal variability by its transverse variability.* |
| *ECG_HRV_PIP* | *Percentage of inflection points of the NN intervals series.* |
| *ECG_HRV_IALS* | *Inverse of the average length of the acceleration/deceleration segments.* |
| *ECG_HRV_PSS* | *Percentage of short segments.* |
| *ECG_HRV_PAS* | *Percentage of NN intervals in alternation segments.* |
| *ECG_HRV_GI* | *Guzik’s Index, defined as the distance of points above line of identity (LI) to LI divided by the distance of all points in Poincaré plot to LI except those that are located on LI.* |
| *ECG_HRV_SI* | *Slope Index, defined as the phase angle of points above LI divided by the phase angle of all points in Poincaré plot except those that are located on LI.* |
| *ECG_HRV_AI* | *Area Index, defined as the cumulative area of the sectors corresponding to the points that are located above LI divided by the cumulative area of sectors corresponding to all points in the Poincaré plot except those that are located on LI.* |
| *ECG_HRV_PI* | *Porta’s Index, defined as the number of points below LI divided by the total number of points in Poincaré plot except those that are located on LI.* |
| *ECG_HRV_C1d* | *Contributions of heart rate decelerations to short-term HRV.* |
| *ECG_HRV_C1a* | *Contributions of heart rate accelerations to short-term HRV.* |
| *ECG_HRV_SD1d* | *Short-term variance of contributions of decelerations (prolongations of NN intervals).* |
| *ECG_HRV_SD1a* | *Short-term variance of contributions of accelerations (shortenings of NN intervals).* |
| *ECG_HRV_C2d* | *Contributions of heart rate decelerations to long-term HRV.* |
| *ECG_HRV_C2a* | *Contributions of heart rate accelerations to long-term HRV.* |
| *ECG_HRV_SD2d* | *Long-term variance of contributions of decelerations (prolongations of NN intervals).* |
| *ECG_HRV_SD2a* | *Long-term variance of contributions of accelerations (shortenings of NN intervals).* |
| *ECG_HRV_Cd* | *Total contributions of heart rate decelerations to HRV.* |
| *ECG_HRV_Ca* | *Total contributions of heart rate accelerations to HRV.* |
| *ECG_HRV_SDNNd* | *Total variance of contributions of decelerations (prolongations of NN intervals).* |
| *ECG_HRV_SDNNa* | *Total variance of contributions of accelerations (shortenings of NN intervals).* |
| *ECG_HRV_DFA_alpha1* | *The monofractal detrended fluctuation analysis of the HR signal, corresponding to short-term correlations.* |
| *ECG_HRV_DFA_alpha2* | *The monofractal detrended fluctuation analysis of the HR signal, corresponding to long-term correlations.* |
| *ECG_HRV_ApEn* | *Approximate Entropy, a measure used to quantify the amount of regularity and unpredictability of fluctuations in a time series.* |
| *ECG_HRV_SampEn* | *Sample Entropy, a measure of the regularity and complexity of a time series.* |
| *ECG_HRV_ShanEn* | *Shannon Entropy, a measure of the disorder or randomness of a system.* |
| *ECG_HRV_FuzzyEn* | *Fuzzy Entropy, a measure of the complexity of a time series.* |
| *ECG_HRV_MSEn* | *Multiscale Entropy, quantifies the complexity of time-series data over multiple temporal scales.* |
| *ECG_HRV_CMSEn* | *Composite Multiscale Entropy, an extension of multiscale entropy, accounting for multiple temporal scales.* |
| *ECG_HRV_RCMSEn* | *Refined Composite Multiscale Entropy, another extension of multiscale entropy.* |
| *ECG_HRV_CD* | *Correlation Dimension, a measure used to describe the amount of space filled by a set of points, indicating the complexity of the data.* |
| *ECG_HRV_HFD* | *Higuchi's Fractal Dimension, used to measure the complexity of a time series.* |
| *ECG_HRV_KFD* | *Katz's Fractal Dimension, quantifies the complexity of a time series by measuring the 'roughness' of its graph.* |
| *ECG_HRV_LZC* | *Lempel-Ziv Complexity, a measure of the complexity of a time series based on the number of distinct substrings and the rate of their occurrence.* |
| *ECG_HRV_MFDFA_alpha1_Width* | *Indices related to the multifractal spectrum for alpha1 corresponding to Width. (alpha1 : corresponding to short-term correlations)* |
| *ECG_HRV_MFDFA_alpha1_Peak* | *Indices related to the multifractal spectrum for alpha1 corresponding to Peak.* |
| *ECG_HRV_MFDFA_alpha1_Mean* | *Indices related to the multifractal spectrum for alpha1 corresponding to Mean.* |
| *ECG_HRV_MFDFA_alpha1_Max* | *Indices related to the multifractal spectrum for alpha1 corresponding to Max.* |
| *ECG_HRV_MFDFA_alpha1_Delta* | *Indices related to the multifractal spectrum for alpha1 corresponding to Delta.* |
| *ECG_HRV_MFDFA_alpha1_Asymmetry* | *Indices related to the multifractal spectrum for alpha1 corresponding to Asymmetry.* |
| *ECG_HRV_MFDFA_alpha1_Fluctuation* | *Indices related to the multifractal spectrum for alpha1 corresponding to Fluctuation.* |
| *ECG_HRV_MFDFA_alpha1_Increment* | *Indices related to the multifractal spectrum for alpha1 corresponding to Increment.* |
| *ECG_HRV_MFDFA_alpha2_Width* | *Indices related to the multifractal spectrum for alpha2 corresponding to Width.(alpha2 : corresponding to long-term correlations)* |
| *ECG_HRV_MFDFA_alpha2_Peak* | *Indices related to the multifractal spectrum for alpha2 corresponding to Peak.* |
| *ECG_HRV_MFDFA_alpha2_Mean* | *Indices related to the multifractal spectrum for alpha2 corresponding to Mean.* |
| *ECG_HRV_MFDFA_alpha2_Max* | *Indices related to the multifractal spectrum for alpha2 corresponding to Max.* |
| *ECG_HRV_MFDFA_alpha2_Delta* | *Indices related to the multifractal spectrum for alpha2 corresponding to Delta.* |
| *ECG_HRV_MFDFA_alpha2_Asymmetry* | *Indices related to the multifractal spectrum for alpha2 corresponding to Asymmetry.* |
| *ECG_HRV_MFDFA_alpha2_Fluctuation* | *Indices related to the multifractal spectrum for alpha2 corresponding to Fluctuation.* |
| *ECG_HRV_MFDFA_alpha2_Increment* | *Indices related to the multifractal spectrum for alpha2 corresponding to Increment.* |

**Supplementary Table 3. Comparison of Simple Logistic Regression Outcomes for Respiratory signals and Breath Rate Variability Data Between In-Shock and Normal Periods**

| **Variables** | **OR (95% CI) ^a^** | **Variables** | **OR (95% CI) ^a^** | |
| --- | --- | --- | --- | --- |
| RESP_RRV_LF | 15.98 (0.09 to 27.58) | RESP_Cycle_Rate_STD | 0.99 (0.98 to 0.99) ^**^ | |
| RESP_Cycle_Symmetry_PeakTrough_Mean | 7.8 (4.08 to 14.93) ^**^ | RESP_Amplitude_skewness | 0.99 (0.96 to 1.02) | |
| RESP_Cycle_Symmetry_PeakTrough_Median | 7.1 (3.92 to 12.88) ^**^ | RESP_Cycle_Rate_Mean | 0.98 (0.97 to 0.99) ^**^ | |
| RESP_Cycle _RVT_Min | 5.61 (2.3 to 13.68) ^**^ | RESP_Cycle_Rate_Median | 0.98 (0.97 to 0.99) ^**^ | |
| RESP_Cycle_Symmetry_PeakTrough_Hurst | 4.62 (2.88 to 7.41) ^**^ | RESP_Cycle_Amplitude_Skewness | 0.98 (0.95 to 1.01) | |
| RESP_Cycle_Symmetry_RiseDecay_Hurst | 3.65 (2.18 to 6.11) ^**^ | RESP_Width_skewness | 0.96 (0.93 to 0.99) ^*^ | |
| RESP_Cycle_Symmetry_RiseDecay_Min | 3.11 (1.67 to 5.78) ^**^ | RESP_Cycle_RVT_STD | 0.96 (0.27 to 3.44) | |
| RESP_Cycle_RVT_Mean | 2.47 (1.5 to 4.07) ^**^ | RESP_RRV_MFDFA_α2_Delta | 0.93 (0.84 to 1.04) | |
| RESP_RRV_LFn | 2.34 (1.51 to 3.62) ^**^ | RESP_Width_interquartile-range | 0.93 (0.82 to 1.05) | |
| RESP_Cycle_RVT_Median | 2.26 (1.4 to 3.65) ^**^ | RESP_Width_median-IQRrate | 0.93 (0.82 to 1.05) | |
| RESP_Cycle_Symmetry_PeakTrough_Min | 2.19 (1.48 to 3.23) ^**^ | RESP_Width_Standard deviation | 0.93 (0.78 to 1.11) | |
| RESP_Cycle_RVT_Hurst | 2.18 (1.4 to 3.39) ^**^ | RESP_RRV_MFDFA_α2_Increment | 0.92 (0.61 to 1.39) | |
| RESP_Cycle_Amplitude_Min | 2.07 (1.58 to 2.7) ^**^ | RESP_Cycle_Symmetry_RiseDecay_Lyapunov | 0.92 (0.01 to 85.29) | |
| RESP_Cycle_Amplitude_Hurst | 2.06 (1.37 to 3.11) ^**^ | RESP_TimePeak_skewness | 0.91 (0.48 to 1.75) ^*^ | |
| RESP_RRV_MFDFA_α1_Peak | 1.58 (1.22 to 2.04) ^**^ | RESP_TimeOnsets_skewness | 0.91 (0.48 to 1.74) | |
| RESP_Amplitude_Mean | 1.51 (1.25 to 1.81) ^**^ | RESP_Cycle_Symmetry_PeakTrough_Skewness | 0.88 (0.84 to 0.92) ^**^ | |
| RESP_Cycle_Amplitude_Mean | 1.45 (1.21 to 1.75) ^**^ | RESP_RRV_MFDFA_α2_Max | 0.87 (0.77 to 0.99) ^*^ | |
| RESP_Cycle_RVT_Max | 1.43 (1.11 to 1.83) ^*^ | RESP_Cycle_Amplitude_SampEn | 0.86 (0.76 to 0.97) ^*^ | |
| RESP_Amplitude_Median | 1.4 (1.18 to 1.66) ^**^ | RESP_Amplitude_Standard deviation | 0.83 (0.5 to 1.4) | |
| RESP_Cycle_Amplitude_Median | 1.36 (1.14 to 1.62) ^**^ | RESP_RRV_MFDFA_α1_Asymmetry | 0.82 (0.54 to 1.26) | |
| RESP_Cycle_Rate_Lyapunov | 1.33 (0.03 to 57.11) | RESP_Cycle_Amplitude_STD | 0.82 (0.48 to 1.39) | |
| RESP_RRV_DFA_α1 | 1.28 (0.85 to 1.91) | RESP_RRV_SampEn | 0.81 (0.7 to 0.91) ^**^ | |
| RESP_Cycle_Rate_Hurst | 1.26 (0.85 to 1.88) | RESP_RRV_ApEn | 0.77 (0.55 to 1.08) | |
| RESP_Cycle_Amplitude_Max | 1.18 (1.05 to 1.32) ^*^ | RESP_Cycle_Symmetry_PeakTrough_SampEn | 0.76 (0.67 to 0.87) ^**^ | |
| RESP_Width_Mean | 1.17 (1.06 to 1.28) ^**^ | RESP_Cycle_RVT_SampEn | 0.76 (0.67 to 0.86) ^**^ | |
| RESP_Width_Median | 1.15 (1.06 to 1.25) ^**^ | RESP_Cycle_Symmetry_RiseDecay_Median | 0.74 (0.39 to 1.4) | |
| RESP_RRV_SD2SD1 | 1.15 (0.92 to 1.43) | RESP_Cycle_Rate_SampEn | 0.73 (0.64 to 0.84) ^**^ | |
| RESP_RRV_MFDFA_α1_Mean | 1.13 (0.95 to 1.34) | RESP_Cycle_Symmetry_RiseDecay_Mean | 0.67 (0.32 to 1.4) | |
| RESP_RRV_MFDFA_α2_Width | 1.12 (0.98 to 1.29) | RESP_RRV_MFDFA_α1_Fluctuation | 0.67 (0 to 2717.44) | |
| RESP_RRV_MFDFA_α2_Mean | 1.11 (0.91 to 1.33) | RESP_RRV_MCVBB | 0.66 (0.47 to 0.92) ^*^ | |
| RESP_RRV_MFDFA_α2_Peak | 1.08 (0.87 to 1.34) | RESP_Amplitude_interquartile-range | 0.63 (0.44 to 0.9) ^*^ | |
| RESP_Cycle_Symmetry_RiseDecay_Kurtosis | 1.06 (1.04 to 1.08) ^**^ | RESP_Amplitude_median-IQRrate | 0.63 (0.44 to 0.9) ^*^ | |
| RESP_RRV_MFDFA_α1_Max | 1.06 (0.97 to 1.16) | RESP_Cycle_Symmetry_RiseDecay_SampEn | 0.61 (0.52 to 0.69) ^**^ | |
| RESP_RRV_DFA_α2 | 1.06 (0.82 to 1.38) | RESP_RRV_CVSD | 0.5 (0.38 to 0.66) ^**^ | |
| RESP_RRV_MFDFA_α2_Asymmetry | 1.05 (0.84 to 1.33) | RESP_RRV_CVBB | 0.49 (0.35 to 0.68) ^**^ | |
| RESP_Cycle_RVT_Skewness | 1.04 (1.01 to 1.08) ^*^ | RESP_RRV_HFn | 0.47 (0.29 to 0.74) ^**^ | |
| RESP_RRV_MFDFA_α1_Width | 1.04 (0.94 to 1.14) | RESP_Cycle_Symmetry_PeakTrough_Max | 0.42 (0.18 to 1) | |
| RESP_RRV_MFDFA_α1_Increment | 1.04 (0.88 to 1.23) | RESP_Cycle_Symmetry_RiseDecay_Max | 0.27 (0.14 to 0.54) ^**^ | |
| RESP_RRV_MFDFA_α1_Delta | 1.03 (0.95 to 1.11) | RESP_Cycle_Symmetry_PeakTrough_Lyapunov | 0.18 (0 to 24.4) | |
| RESP_RRV_MeanBB | 1.02 (1.01 to 1.03) ^**^ | RESP_Cycle_Symmetry_PeakTrough_STD | 0.16 (0.04 to 0.63) ^*^ | |
| RESP_RRV_MadBB | 1.02 (1.01 to 1.03) | RESP_Cycle_Symmetry_RiseDecay_STD | 0.09 (0.02 to 0.38) ^**^ | |
| RESP_RRV_SD1 | 1.02 (1.01 to 1.03) | RESP_Cycle_Amplitude_Lyapunov | 0.06 (0 to 8.27) | |
| RESP_RRV_SDSD | 1.02 (1.01 to 1.03) | RESP_RRV_MFDFA_α2_Fluctuation | 0.05 (0 to 3.07) | |
| RESP_TimeOnsets_Median | 1.02 (1.01 to 1.03) | RESP_RRV_VLF | 0.03 (0.01 to 0.23) ^*^ | |
| RESP_TimePeak_median-IQRrate | 1.02 (1.01 to 1.03) | RESP_Cycle_RVT_Lyapunov | 0.03 (0 to 3.49) | |
| RESP_Cycle_Rate_Skewness | 1.02 (0.99 to 1.06) | RESP_RRV_HF | 0.01 (0 to 0.01) ^**^ | |
| RESP_Cycle_Symmetry_RiseDecay_Skewness | 1.02 (0.95 to 1.1) |  |  | |
| RESP_TimePeak_Mean | 1.01 (1.01 to 1.02) ^**^ |  |  | |
| RESP_TimePeak_Median | 1.01 (1.01 to 1.02) ^**^ |  |  | |
| RESP_TimeOnsets_interquartile-range | 1.01 (1.01 to 1.02) |  |  | |
| RESP_TimeOnsets_Mean | 1.01 (1.01 to 1.02) |  |  | |
| RESP_RRV_MedianBB | 1.01 (1 to 1.02) ^**^ |  |  | |
| RESP_RRV_LFHF | 1.01 (1 to 1.02) |  |  | |
| RESP_RRV_RMSSD | 1.01 (1 to 1.02) |  |  | |
| RESP_RRV_SD2 | 1.01 (1 to 1.02) |  |  | |
| RESP_RRV_SDBB | 1.01 (1 to 1.02) |  |  | |
| RESP_TimeOnsets_median-IQRrate | 1.01 (1 to 1.02) |  |  | |
| RESP_TimePeak_interquartile-range | 1.01 (1 to 1.02) |  |  | |
| RESP_Cycle_Amplitude_Kurtosis | 1.01 (1 to 1.01) ^**^ |  |  | |
| RESP_Cycle_Rate_Kurtosis | 1.01 (1 to 1.01) ^*^ |  |  | |
| RESP_Cycle_RVT_Kurtosis | 1.01 (1 to 1.01) ^*^ |  |  | |
| RESP_Cycle_Symmetry_PeakTrough_Kurtosis | 1.01 (1 to 1.01) ^*^ |  |  | |
| RESP_Cycle_Rate_Max | 1.01 (0.99 to 1.03) ^**^ |  |  | |
| RESP_TimeOnsets_SD | 1.01 (0.99 to 1.01) |  |  | |
| RESP_TimePeak_ STD | 1.01 (0.99 to 1.01) |  |  | |
| RESP_CYCLE_Rate_Min | 1.01 (0.98 to 1.02) |  |  | |
| Multivariable logistic regression models adjusted for age, sex, body mass index, neck and waist circumference.  **p* < 0.05; ***p* < 0.01. | | | |  |

| *Definition of abbreviations:* | | | |
| --- | --- | --- | --- |
| *Feature Name* | *Feature Description* | *Metric* | *Metric Description* |
| *RESP_Amplitude_Mean* | *Amplitudes between peaks and troughs* | *Mean* | *Mean value* |
| *RESP_Amplitude_Median* | *Amplitudes between peaks and troughs* | *Median* | *Median value* |
| *RESP_Amplitude_interquartile-range* | *Amplitudes between peaks and troughs* | *interquartile-range* | *Interquartile Range (IQR)* |
| *RESP_Amplitude_median-IQRrate* | *Amplitudes between peaks and troughs* | *median-IQRrate* | *Rate of Median to IQR* |
| *RESP_Amplitude_Standard deviation* | *Amplitudes between peaks and troughs* | *Standard deviation* | *Standard Deviation* |
| *RESP_Amplitude_skewness* | *Amplitudes between peaks and troughs* | *skewness* | *Skewness* |
| *RESP_Width_Mean* | *Time intervals between peaks and troughs* | *Mean* | *Mean value* |
| *RESP_Width_Median* | *Time intervals between peaks and troughs* | *Median* | *Median value* |
| *RESP_Width_interquartile-range* | *Time intervals between peaks and troughs* | *interquartile-range* | *Interquartile Range (IQR)* |
| *RESP_Width_median-IQRrate* | *Time intervals between peaks and troughs* | *median-IQRrate* | *Rate of Median to IQR* |
| *RESP_Width_Standard deviation* | *Time intervals between peaks and troughs* | *Standard deviation* | *Standard Deviation* |
| *RESP_Width_skewness* | *Time intervals between peaks and troughs* | *skewness* | *Skewness* |
| *RESP_TimePeak_Mean* | *Time intervals between peaks* | *Mean* | *Mean value* |
| *RESP_TimePeak_Median* | *Time intervals between peaks* | *Median* | *Median value* |
| *RESP_TimePeak_interquartile-range* | *Time intervals between peaks* | *interquartile-range* | *Interquartile Range (IQR)* |
| *RESP_TimePeak_median-IQRrate* | *Time intervals between peaks* | *median-IQRrate* | *Rate of Median to IQR* |
| *RESP_TimePeak_Standard deviation* | *Time intervals between peaks* | *Standard deviation* | *Standard Deviation* |
| *RESP_TimePeak_skewness* | *Time intervals between peaks* | *skewness* | *Skewness* |
| *RESP_TimeOnsets_Mean* | *Time intervals between troughs* | *Mean* | *Mean value* |
| *RESP_TimeOnsets_Median* | *Time intervals between troughs* | *Median* | *Median value* |
| *RESP_TimeOnsets_interquartile-range* | *Time intervals between troughs* | *interquartile-range* | *Interquartile Range (IQR)* |
| *RESP_TimeOnsets_median-IQRrate* | *Time intervals between troughs* | *median-IQRrate* | *Rate of Median to IQR* |
| *RESP_TimeOnsets_Standard deviation* | *Time intervals between troughs* | *Standard deviation* | *Standard Deviation* |
| *RESP_TimeOnsets_skewness* | *Time intervals between troughs* | *skewness* | *Skewness* |
| *Feature Code* | *Feature Description* | *Metric* | *Metric Description* |
| *RESP_Amplitude_Min* | *Amplitude of the cycle (average voltage difference between the trough and adjacent peaks)* | *Min* | *Minimum value* |
| *RESP_Amplitude_Max* | *Amplitude of the cycle (average voltage difference between the trough and adjacent peaks)* | *Max* | *Maximum value* |
| *RESP_Amplitude_Mean* | *Amplitude of the cycle (average voltage difference between the trough and adjacent peaks)* | *Mean* | *Average value* |
| *RESP_Amplitude_Median* | *Amplitude of the cycle (average voltage difference between the trough and adjacent peaks)* | *Median* | *Median value* |
| *RESP_Amplitude_STD* | *Amplitude of the cycle (average voltage difference between the trough and adjacent peaks)* | *STD* | *Standard Deviation* |
| *RESP_Amplitude_Skewness* | *Amplitude of the cycle (average voltage difference between the trough and adjacent peaks)* | *Skewness* | *Skewness* |
| *RESP_Amplitude_Kurtosis* | *Amplitude of the cycle (average voltage difference between the trough and adjacent peaks)* | *Kurtosis* | *Kurtosis* |
| *RESP_Amplitude_Hurst* | *Amplitude of the cycle (average voltage difference between the trough and adjacent peaks)* | *Hurst* | *Hurst exponent* |
| *RESP_Amplitude_Lyapunov* | *Amplitude of the cycle (average voltage difference between the trough and adjacent peaks)* | *Lyapunov* | *Lyapunov exponent* |
| *RESP_Amplitude_SampEn* | *Amplitude of the cycle (average voltage difference between the trough and adjacent peaks)* | *SampEn* | *Sample Entropy* |
| *RESP_Rate_Min* | *Rate of respiratory cycles* | *Min* | *Minimum value* |
| *RESP_Rate_Max* | *Rate of respiratory cycles* | *Max* | *Maximum value* |
| *RESP_Rate_Mean* | *Rate of respiratory cycles* | *Mean* | *Average value* |
| *RESP_Rate_Median* | *Rate of respiratory cycles* | *Median* | *Median value* |
| *RESP_Rate_STD* | *Rate of respiratory cycles* | *STD* | *Standard Deviation* |
| *RESP_Rate_Skewness* | *Rate of respiratory cycles* | *Skewness* | *Skewness* |
| *RESP_Rate_Kurtosis* | *Rate of respiratory cycles* | *Kurtosis* | *Kurtosis* |
| *RESP_Rate_Hurst* | *Rate of respiratory cycles* | *Hurst* | *Hurst exponent* |
| *RESP_Rate_Lyapunov* | *Rate of respiratory cycles* | *Lyapunov* | *Lyapunov exponent* |
| *RESP_Rate_SampEn* | *Rate of respiratory cycles* | *SampEn* | *Sample Entropy* |
| *RESP_RVT_Min* | *Respiratory Volume per Time (product of respiratory volume and breathing rate)* | *Min* | *Minimum value* |
| *RESP_RVT_Max* | *Respiratory Volume per Time (product of respiratory volume and breathing rate)* | *Max* | *Maximum value* |
| *RESP_RVT_Mean* | *Respiratory Volume per Time (product of respiratory volume and breathing rate)* | *Mean* | *Average value* |
| *RESP_RVT_Median* | *Respiratory Volume per Time (product of respiratory volume and breathing rate)* | *Median* | *Median value* |
| *RESP_RVT_STD* | *Respiratory Volume per Time (product of respiratory volume and breathing rate)* | *STD* | *Standard Deviation* |
| *RESP_RVT_Skewness* | *Respiratory Volume per Time (product of respiratory volume and breathing rate)* | *Skewness* | *Skewness* |
| *RESP_RVT_Kurtosis* | *Respiratory Volume per Time (product of respiratory volume and breathing rate)* | *Kurtosis* | *Kurtosis* |
| *RESP_RVT_Hurst* | *Respiratory Volume per Time (product of respiratory volume and breathing rate)* | *Hurst* | *Hurst exponent* |
| *RESP_RVT_Lyapunov* | *Respiratory Volume per Time (product of respiratory volume and breathing rate)* | *Lyapunov* | *Lyapunov exponent* |
| *RESP_RVT_SampEn* | *Respiratory Volume per Time (product of respiratory volume and breathing rate)* | *SampEn* | *Sample Entropy* |
| *RESP_Symmetry_PeakTrough_Min* | *Symmetry (relative amount of a cycle between rise midpoint and subsequent decay midpoint)* | *Min* | *Minimum value* |
| *RESP_Symmetry_PeakTrough_Max* | *Symmetry (relative amount of a cycle between rise midpoint and subsequent decay midpoint)* | *Max* | *Maximum value* |
| *RESP_Symmetry_PeakTrough_Mean* | *Symmetry (relative amount of a cycle between rise midpoint and subsequent decay midpoint)* | *Mean* | *Average value* |
| *RESP_Symmetry_PeakTrough_Median* | *Symmetry (relative amount of a cycle between rise midpoint and subsequent decay midpoint)* | *Median* | *Median value* |
| *RESP_Symmetry_PeakTrough_STD* | *Symmetry (relative amount of a cycle between rise midpoint and subsequent decay midpoint)* | *STD* | *Standard Deviation* |
| *RESP_Symmetry_PeakTrough_Skewness* | *Symmetry (relative amount of a cycle between rise midpoint and subsequent decay midpoint)* | *Skewness* | *Skewness* |
| *RESP_Symmetry_PeakTrough_Kurtosis* | *Symmetry (relative amount of a cycle between rise midpoint and subsequent decay midpoint)* | *Kurtosis* | *Kurtosis* |
| *RESP_Symmetry_PeakTrough_Hurst* | *Symmetry (relative amount of a cycle between rise midpoint and subsequent decay midpoint)* | *Hurst* | *Hurst exponent* |
| *RESP_Symmetry_PeakTrough_Lyapunov* | *Symmetry (relative amount of a cycle between rise midpoint and subsequent decay midpoint)* | *Lyapunov* | *Lyapunov exponent* |
| *RESP_Symmetry_PeakTrough_SampEn* | *Symmetry (relative amount of a cycle between rise midpoint and subsequent decay midpoint)* | *SampEn* | *Sample Entropy* |
| *RESP_Symmetry_RiseDecay_Min* | *Symmetry (fraction of the period that the cycle is in the rise phase)* | *Min* | *Minimum value* |
| *RESP_Symmetry_RiseDecay_Max* | *Symmetry (fraction of the period that the cycle is in the rise phase)* | *Max* | *Maximum value* |
| *RESP_Symmetry_RiseDecay_Mean* | *Symmetry (fraction of the period that the cycle is in the rise phase)* | *Mean* | *Average value* |
| *RESP_Symmetry_RiseDecay_Median* | *Symmetry (fraction of the period that the cycle is in the rise phase)* | *Median* | *Median value* |
| *RESP_Symmetry_RiseDecay_STD* | *Symmetry (fraction of the period that the cycle is in the rise phase)* | *STD* | *Standard Deviation* |
| *RESP_Symmetry_RiseDecay_Skewness* | *Symmetry (fraction of the period that the cycle is in the rise phase)* | *Skewness* | *Skewness* |
| *RESP_Symmetry_RiseDecay_Kurtosis* | *Symmetry (fraction of the period that the cycle is in the rise phase)* | *Kurtosis* | *Kurtosis* |
| *RESP_Symmetry_RiseDecay_Hurst* | *Symmetry (fraction of the period that the cycle is in the rise phase)* | *Hurst* | *Hurst exponent* |
| *RESP_Symmetry_RiseDecay_Lyapunov* | *Symmetry (fraction of the period that the cycle is in the rise phase)* | *Lyapunov* | *Lyapunov exponent* |
| *RESP_Symmetry_RiseDecay_SampEn* | *Symmetry (fraction of the period that the cycle is in the rise phase)* | *SampEn* | *Sample Entropy* |

| ***Feature*** | ***Description*** |
| --- | --- |
| *RESP_RRV_RMSSD* | *The root mean square of successive differences of the breath-to-breath intervals.* |
| *RESP_RRV_MeanBB* | *The mean value of the breath-to-breath intervals.* |
| *RESP_RRV_SDBB* | *The standard deviation of the breath-to-breath intervals.* |
| *RESP_RRV_SDSD* | *The standard deviation of the successive differences between adjacent breath-to-breath intervals.* |
| *RESP_RRV_CVBB* | *The Coefficient of Variation (CV) of the breath-to-breath intervals.* |
| *RESP_RRV_CVSD* | *The ratio of RMSSD to the average of the breath-to-breath intervals.* |
| *RESP_RRV_MedianBB* | *The median of the breath-to-breath intervals.* |
| *RESP_RRV_MadBB* | *The Median Absolute Deviation (MAD) of the breath-to-breath intervals.* |
| *RESP_RRV_MCVBB* | *The ratio of MAD to the median of the breath-to-breath intervals.* |
| *RESP_RRV_VLF* | *Spectral power density pertaining to very low frequency band (i.e., 0 to .04 Hz) by default.* |
| *RESP_RRV_LF* | *Spectral power density pertaining to low frequency band (i.e., .04 to .15 Hz) by default.* |
| *RESP_RRV_HF* | *Spectral power density pertaining to high frequency band (i.e., .15 to .4 Hz) by default.* |
| *RESP_RRV_LFHF* | *The ratio of low frequency power to high frequency power.* |
| *RESP_RRV_LFn* | *The normalized low frequency, obtained by dividing the low frequency power by the total power.* |
| *RESP_RRV_HFn* | *The normalized high frequency, obtained by dividing the low frequency power by total power.* |
| *RESP_RRV_SD1* | *SD1 is a measure of the spread of breath-to-breath intervals on the Poincar? plot perpendicular to the line of identity. It is an index of short-term variability.* |
| *RESP_RRV_SD2* | *SD2 is a measure of the spread of breath-to-breath intervals on the Poincar? plot along the line of identity. It is an index of long-term variability.* |
| *RESP_RRV_SD2SD1* | *The ratio between short and long term fluctuations of the breath-to-breath intervals (SD2 divided by SD1).* |
| *RESP_RRV_ApEn* | *The approximate entropy.* |
| *RESP_RRV_SampEn* | *The sample entropy.* |
| *RESP_RRV_DFA_alpha1* | *the "short-term" fluctuation value generated from Detrended Fluctuation Analysis i.e. the root mean square deviation from the fitted trend of the breath-to-breath intervals. Will only be computed if more than 160 breath cycles in the signal.* |
| *RESP_RRV_DFA_alpha2* | *the long-term fluctuation value. Will only be computed if more than 640 breath cycles in the signal.* |
| *RESP_RRV_MFDFA_alpha1_Width* | *Multifractality degree. (MF-DFA is an extension of DFA, used to explore the multifractal fluctuation value of time series data. Alpha1 means will only be computed if more than 160 breath cycles in the signal.)* |
| *RESP_RRV_MFDFA_alpha1_Peak* | *A measure of the self-affinity of the signal, and a high value is an indicator of high degree of correlation between the data points.* |
| *RESP_RRV_MFDFA_alpha1_Mean* | *The average fluctuations of the signal.* |
| *RESP_RRV_MFDFA_alpha1_Max* | *The maximum fluctuation of the signal.* |
| *RESP_RRV_MFDFA_alpha1_Delta* | *The range of fluctuations of the signal.* |
| *RESP_RRV_MFDFA_alpha1_Asymmetry* | *The centrality of the peak of the spectrum.* |
| *RESP_RRV_MFDFA_alpha1_Fluctuation* | *The power of the second derivative of h(q).* |
| *RESP_RRV_MFDFA_alpha1_Increment* | *Robustness of Hurst’s exponent distribution.* |
| *RESP_RRV_MFDFA_alpha2_Width* | *Multifractality degree (RRV_MFDFA _alpha2 : the long-term multifractal fluctuation value. Will only be computed if more than 640 breath cycles in the signal.)* |
| *RESP_RRV_MFDFA_alpha2_Peak* | *A measure of the self-affinity of the signal, and a high value is an indicator of high degree of correlation between the data points.* |
| *RESP_RRV_MFDFA_alpha2_Mean* | *The average fluctuations of the signal.* |
| *RESP_RRV_MFDFA_alpha2_Max* | *The maximum fluctuation of the signal.* |
| *RESP_RRV_MFDFA_alpha2_Delta* | *The range of fluctuations of the signal.* |
| *RESP_RRV_MFDFA_alpha2_Asymmetry* | *The centrality of the peak of the spectrum.* |
| *RESP_RRV_MFDFA_alpha2_Fluctuation* | *The power of the second derivative of h(q).* |
| *RESP_RRV_MFDFA_alpha2_Increment* | *Robustness of Hurst’s exponent distribution.* |

**Supplementary Table 4. Comparison of Simple Logistic Regression Outcomes for Peripheral Capillary Oxygen Saturation Data Between In-Shock and Normal Periods**

| **Variables** | **OR (95% CI) ^a^** | **Variables** | **OR (95% CI) ^a^** |
| --- | --- | --- | --- |
| SpO2_CTM | 1.41 (0.65 to 3.06) | SpO2_Min | 0.98 (0.97 to 1.01) * |
| SpO2_SampEn | 1.06 (0.43 to 2.59) | SpO2_Median | 0.95 (0.93 to 0.98) ** |
| SpO2_STD | 1.06 (0.98 to 1.15) | SpO2_Mean | 0.95 (0.93 to 0.98) ** |
|  |  | SpO2_Max | 0.93 (0.9 to 0.96) ** |
|  |  | SpO2_LZC | 0.42 (0.21 to 0.81) * |
| Definition of abbreviations:  Multivariable logistic regression models adjusted for age, sex, body mass index, neck and waist circumference.  **p* < 0.05; ***p* < 0.01. | | | |

| ***Feature*** | ***Description*** |
| --- | --- |
| *SpO2_Mean* | *Average value of all the SpO2 readings in a given set.* |
| *SpO2_STD* | *Measures the amount of variability or dispersion of the SpO2 readings.* |
| *SpO2_Median* | *Middle value when all the SpO2 readings are arranged in order.* |
| *SpO2_Min* | *Smallest value in a given set of SpO2 readings.* |
| *SpO2_Max* | *Highest value in a given set of SpO2 readings.* |
| *SpO2_SampEn* | *Sample entropy : Quantifies the unpredictability of fluctuations in the SpO2 readings. A higher value indicates more complexity and less predictability, while a lower value suggests more regular and predictable fluctuations.* |
| *SpO2_LZC* | *Lempel-Ziv complexity : A non-linear method used to quantify the complexity or randomness of a data series. A higher value indicates more complexity in the SpO2 signal, while a lower one suggests a more regular or repetitive signal.* |
| *SpO2_CTM* | *Central Tendency Measure : Measures the tendency of the SpO2 readings to cluster around a central value. A value close to 1 indicates a strong central tendency, while a value far from 1 suggests a more dispersed distribution.* |

**Supplementary Table 5.** **Confusion Matrix in Training, Validation, and Testing Datasets** **for Established Models**

| **Models** | **True Positives** | **False Negatives** | **False Positives** | **True Negatives** |
| --- | --- | --- | --- | --- |
| **Training and validation set** | | | | |
| Weighted_Ensemble | 261 | 50 | 43 | 267 |
| CatBoost | 256 | 55 | 51 | 260 |
| LightGBM | 260 | 51 | 38 | 263 |
| RF | 251 | 60 | 45 | 265 |
| XGBoost | 256 | 55 | 52 | 259 |
| ET | 241 | 70 | 50 | 261 |
| **Testing set** | | | | |
| Weighted_Ensemble | 62 | 16 | 8 | 69 |
| CatBoost | 61 | 17 | 9 | 68 |
| LightGBM | 58 | 20 | 9 | 68 |
| RF | 61 | 17 | 10 | 68 |
| XGBoost | 62 | 16 | 9 | 68 |
| ET | 60 | 18 | 10 | 68 |
| Abbreviation: Weighted_Ensemble: Constructing ensembles from all models; CatBoost: Categorical Boosting; LightGBM: Light gradient boosting machine; RF: Random Forest; XGBoost: Extreme gradient boosting; ET: Extremely randomized trees. | | | | |

**Supplementary Figure 1. Distributions of processed R-R intervals (RRI) derived from processed heart rate (HR) signals**

To eliminate implausible outliers caused by "peak-picking" artifacts, peak interpolation was applied when HR fell below or exceeded 150 bpm. The resulting average HR values corresponded to RRI within the acceptable range (400 milliseconds < RRI < 1500 milliseconds), indicating the suitability of the processed signals for heart rate variability analysis. The average RRI was 708.64 milliseconds, with a standard deviation (SD) of 144.12 milliseconds.

Abbreviation: ms: millisecond

**
